# A pleiotropic recurrent dominant *ITPR3* variant causes a complex multisystemic disease

**DOI:** 10.1126/sciadv.ado5545

**Published:** 2024-09-13

**Authors:** Anne Molitor, Alexandre Lederle, Mirjana Radosavljevic, Vinay Sapuru, Megan E. Zavorka Thomas, Jianying Yang, Mahsa Shirin, Virginie Collin-Bund, Katerina Jerabkova-Roda, Zhichao Miao, Alice Bernard, Véronique Rolli, Pierre Grenot, Carla Noemi Castro, Michelle Rosenzwajg, Elyssa G. Lewis, Richard Person, Uxía-Saraiva Esperón-Moldes, Milja Kaare, Pekka T. Nokelainen, Nurit Assia Batzir, Gal Zaks Hoffer, Nicodème Paul, Tristan Stemmelen, Lydie Naegely, Antoine Hanauer, Sabrina Bibi-Triki, Sarah Grün, Sophie Jung, Ignacio Busnelli, Kornelia Tripolszki, Ruslan Al-Ali, Natalia Ordonez, Peter Bauer, Eunkyung Song, Kristin Zajo, Santiago Partida-Sanchez, Frank Robledo-Avila, Attila Kumanovics, Yoram Louzoun, Aurélie Hirschler, Angélique Pichot, Ori Toker, Cesar Andrés Muñoz Mejía, Nima Parvaneh, Esther Knapp, Joseph H. Hersh, Heather Kenney, Ottavia M. Delmonte, Luigi D. Notarangelo, Jacky G. Goetz, Samir B. Kahwash, Christine Carapito, Rajinder P. S. Bajwa, Caroline Thomas, Stephan Ehl, Bertrand Isidor, Raphael Carapito, Roshini S. Abraham, Richard K. Hite, Nufar Marcus, Aida Bertoli-Avella, Seiamak Bahram

**Affiliations:** ^1^ Laboratoire d’ImmunoRhumatologie Moléculaire, Institut national de la santé et de la recherche médicale (INSERM) UMR_S 1109, Plateforme GENOMAX, Centre de Recherche d’Immunologie et d’Hématologie and Centre de Recherche en Biomédecine de Strasbourg (CRBS), Faculté de Médecine, Fédération Hospitalo-Universitaire OMICARE, Fédération de Médecine Translationnelle de Strasbourg (FMTS), Université de Strasbourg, Strasbourg, France.; ^2^ Institut Thématique Interdisciplinaire (ITI) Transplantex NG de Médecine de Précision de Strasbourg, Faculté de Médecine, Université de Strasbourg, Strasbourg, France.; ^3^ Laboratoire d’Immunologie, Plateau Technique de Biologie, Pôle de Biologie, Nouvel Hôpital Civil, Hôpitaux Universitaires de Strasbourg, Strasbourg, France.; ^4^ Structural Biology Program, Memorial Sloan Kettering Cancer Center, New York, NY, USA.; ^5^ Physiology, Biophysics, and Systems Biology (PBSB) Program, Weill Cornell Graduate School of Biomedical Sciences, New York, NY, USA.; ^6^ Department of Pathology and Laboratory Medicine, Nationwide Children’s Hospital, Columbus, OH, USA.; ^7^ Equipe labellisée, Ligue nationale Contre le Cancer, Strasbourg, France.; ^8^ Guangzhou National Laboratory, Guangzhou International Bio-Island, Guangzhou, China.; ^9^ Translational Research Institute of Brain and Brain-Like Intelligence and Department of Anesthesiology, Shanghai Fourth People's Hospital Affiliated to Tongji University School of Medicine, Shanghai, China.; ^10^ Institute for Immunodeficiency, Center for Chronic Immunodeficiency, Medical Center–University of Freiburg, Faculty of Medicine, University of Freiburg, Freiburg, Germany.; ^11^ Assistance Publique Hôpitaux de Paris, Hôpital Pitié-Salpêtrière, Clinical Investigation Center for Biotherapies (CIC-BTi) and Immunology-Inflammation-Infectiology and Dermatology Department (3iD), Paris, France.; ^12^ Sorbonne Université, INSERM UMR_S 959, Immunology-Immunopathology-Immunotherapy (i3), Paris, France.; ^13^ Norton Children’s Medical Group, University of Louisville School of Medicine, Louisville, KY, USA.; ^14^ GeneDx, Gaithersburg, MD, USA.; ^15^ Blueprint Genetics, A Quest Diagnostics Company, Espoo, Finland.; ^16^ Pediatric Genetics Unit, Schneider Children's Medical Center of Israel, Petach Tikvah, Israel.; ^17^ Faculty of Biology, University of Freiburg, Freiburg, Germany.; ^18^ Centre de Référence des maladies rares orales et dentaires (O-Rares), Pôle de Médecine et de Chirurgie bucco-dentaires, Hôpitaux Universitaires de Strasbourg, Strasbourg, France.; ^19^ CENTOGENE GmbH, Rostock, Germany.; ^20^ Division of Infectious Diseases and Host Defense, Department of Pediatrics, Nationwide Children’s Hospital, Columbus, OH, USA.; ^21^ Institute of Genomic Medicine, Nationwide Children’s Hospital, Columbus, OH, USA.; ^22^ Center for Microbial Pathogenesis, Abigail Wexner Research Institute, Nationwide Children’s Hospital, Columbus, OH, USA.; ^23^ Department of Laboratory Medicine and Pathology, Mayo Clinic, Rochester, MN USA.; ^24^ Department of Mathematics, Bar-Ilan University, Ramat Gan, Israel.; ^25^ Laboratoire de Spectrométrie de Masse Bio-Organique (LSMBO), Institut Pluridisciplinaire Hubert Curien (IPHC), UMR 7178, Université de Strasbourg, CNRS, Infrastructure Nationale de Protéomique ProFI - FR2048, Strasbourg, France.; ^26^ Allergy and Immunology Unit, Shaare Zedek Medical Center, Jerusalem, Israel.; ^27^ Faculty of Medicine Hebrew university, Jerusalem, Israel.; ^28^ Hospital Infantil Napoleón Franco Pareja, Cartagena de Indias, Bolívar, Colombia.; ^29^ Department of Pediatrics, Children's Medical Center, Tehran University of Medical Sciences, Tehran, Iran.; ^30^ Laboratory of Clinical Immunology and Microbiology (LCIM), National Institute of Allergy and Infectious Diseases (NIAID), National Institutes of Health (NIH), Bethesda, MD, USA.; ^31^ Division of Pediatric Hematology, Oncology and Bone Marrow Transplantation, Department of Pediatrics, Nationwide Children’s Hospital, Columbus, OH, USA.; ^32^ Service d'Oncologie-Hématologie et Immunologie Pédiatrique, Hôpital Enfant-Adolescent, CHU Nantes, Nantes, France.; ^33^ Service de Génétique Médicale, Hôpital Hôtel-Dieu, CHU de Nantes, Nantes, France.; ^34^ Allergy and Immunology Unit, Kipper Institute of Immunology, Schneider Children’s Medical Center of Israel, Petah Tikva, Israel.; ^35^ Sackler Faculty of Medicine, Tel Aviv University, Tel Aviv, Israel.; ^36^ The Jeffrey Modell Foundation Israeli Network for Primary Immunodeficiency, New York, NY, USA.

## Abstract

Inositol 1,4,5-trisphosphate (IP3) receptor type 1 (*ITPR1*), *2* (*ITPR2*), and *3* (*ITPR3*) encode the IP3 receptor (IP3R), a key player in intracellular calcium release. In four unrelated patients, we report that an identical *ITPR3* de novo variant—NM_002224.3:c.7570C>T, p.Arg2524Cys—causes, through a dominant-negative effect, a complex multisystemic disorder with immunodeficiency. This leads to defective calcium homeostasis, mitochondrial malfunction, CD4^+^ lymphopenia, a quasi-absence of naïve CD4^+^ and CD8^+^ cells, an increase in memory cells, and a distinct TCR repertoire. The calcium defect was recapitulated in Jurkat knock-in. Site-directed mutagenesis displayed the exquisite sensitivity of Arg^2524^ to any amino acid change. Despite the fact that all patients had severe immunodeficiency, they also displayed variable multisystemic involvements, including ectodermal dysplasia, Charcot-Marie-Tooth disease, short stature, and bone marrow failure. In conclusion, unlike previously reported *ITPR1-3* deficiencies leading to narrow, mainly neurological phenotypes, a recurrent dominant *ITPR3* variant leads to a multisystemic disease, defining a unique role for IP3R3 in the tetrameric IP3R complex.

## Teaser

A de novo variant in the Ca^2+^ modulator *ITPR3* causes a pleiotropic multisystemic disease where immunodeficiency is paramount.

## INTRODUCTION

Cytosolic Ca^2+^ homeostasis is critical for a plethora of fundamental physiological functions, including cell motility, neuronal transmission, gene transcription, cell proliferation, and cell death (*1*). In cells of the immune system in particular, Ca^2+^ influx is regulated by the store-operated Ca^2+^ entry (SOCE) through the calcium release–activated calcium (CRAC) channels (*2*). In lymphocytes, this process is triggered upon engagement of cell surface antigen receptors. This leads to the activation of the membrane-associated phospholipase C-γ1, which subsequently cleaves phosphatidylinositol 4,5-bisphosphate into the membrane-bound diacylglycerol and the soluble inositol 1,4,5-trisphosphate (IP3). The latter then reaches and gates the cognate IP3 receptor (IP3R) at the surface of the endoplasmic reticulum (ER), the main intracellular Ca^2+^ storage site (*3*). IP3R is a homo- or heterotetramer of three highly homologous components encoded by the *IP3R type 1* (*ITPR1*), *2* (*ITPR2*), and *3* (*ITPR3*) genes, respectively, located on human chromosomes 3p26.1, 12p11.23, and 6p21.31 (*4*, *5*). Upon binding of IP3 to IP3R, Ca^2+^ is released from the ER lumen to the cytosol, given its massive existing gradient between the two milieus ([Ca^2+^]_ER_ ~ 0.5 to 1 mM versus [Ca^2+^]_Cyt_ 0.1 μM). The subsequent decrease in intra-ER calcium concentrations activates in turn the ER-embedded “stromal interaction molecules” 1 (STIM1) and 2 (STIM2), EF-hand domain-containing proteins, which sense the decrease in [Ca^2+^]_ER_, oligomerize, and physically bind and activate the plasma membrane-bound CRAC channel ORAI1 (alternatively called “calcium release–activated calcium modulator 1”) (*6*). ORAI1 is a plasma membrane-bound tetraspan protein that acts as a calcium pore of the CRAC channel and allows ion entry inside the cell, replenishing its stores. Two distinct sets of CRAC channelopathies caused by recessive loss-of-function (LoF) variants in *ORAI1* and *STIM1* have been reported in multiple patients (*7*). These lead to a complex life-threatening syndromic combined immunodeficiency with symptomatology including recurrent severe infections, hepatosplenomegaly, autoimmune manifestations, anhidrotic ectodermal dysplasia, and myopathy, which can only be alleviated through allogeneic hematopoietic cell transplantation (HCT). It is of note, however, that in these channelopathies, major lymphoid and myeloid subpopulations are numerically normal (like in a number of other severe combined immunodeficiencies) but functionally impaired, to an extent not completely understood (*8*).

In Online Mendelian Inheritance in Man (OMIM), *ITPR3* is related to autosomal dominant Charcot-Marie-Tooth disease, demyelinating, type 1J (#620111) (*9*, *10*), although the evidence for this association is still limited (as per ClinGen Clinical Validity Framework guidelines) (*11*). Recently, Neumann and colleagues (*12*) reported compound heterozygous *ITPR3* variants in two patients with immune-related phenotypes.

Here, we report that an identical dominant-negative variant in *ITPR3* in four independent families from France, Israel, and the United States is causative of a complex syndromic immunodeficiency with variable multisystemic manifestations. The pleiotropy of this variant helps differentiate the role of IP3R type 3 (IP3R3) within the IP3R complex, as all other *ITPR1-3* variants reported to date cause a rather narrow, mainly neurological, phenotype.

## RESULTS

### A multisystemic clinical phenotype

**Fig. 1.**
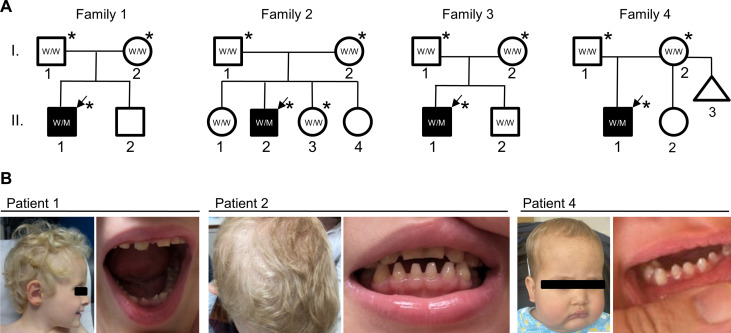
An identical de novo pathogenic *ITPR3* variant in four independent families. (**A**) Pedigrees of four affected families suffering from a complex immunodeficiency syndrome in childhood. Generations are designated by Roman numerals and subjects by Arabic numerals. Squares: male subjects; circles: female subjects; triangle: miscarriage; filled (black) symbols indicate patients, while unfilled (white) symbols indicate unaffected family members. Arrows denote probands in each family. Stars indicate individuals subjected to WES. All probands were heterozygous, and the parents and the tested unaffected siblings were wild type (WT) (W: WT allele, M: mutant/variant allele). (**B**) Ectodermal dysplasia: In patient 1 (left two pictures), sparse and thin scalp hair, sparse and hypopigmented eyebrows, dysmorphic cone-shaped mandibular primary incisors, and lastly the presence of diastema between the teeth is shown (see also an orthopantomogram as well as additional pictures of the patient in fig. S1A). Patient 2 (middle two pictures) displays sparse/thin hair and xerosis (further pictures are available in fig. S1B). One further notes an abnormal shape for teeth, e.g., cone-shaped primary incisors (except maxillary central incisors), and the presence of diastema between the teeth. Patient 4 (right two pictures) had similar anomalies to those of other patients. Additional pictures in fig. S1.

Patient 1 is an 8-year-old boy born at term and is the second child (his 11-year-old brother is presently healthy) of unrelated parents of French origin (Fig. 1A, family 1). The clinical symptoms started with severe bronchiolitis that led to a first hospital admission at age of 3.5 months. At age of 7.5 months, he presented with severe varicella with generalized cutaneous, mucosal, and pulmonary lesions (and possible encephalopathy), necessitating admission to the pediatric intensive care unit (PICU) for 10 days. This was followed by severe stomatitis (at age 19 months), followed by severe bocavirus-associated pneumonia 5 months later, both necessitating readmission to the PICU. By 2 years of age, he had infectious mononucleosis, paronychia of a toenail, and herpes zoster, leading to additional hospitalizations. In addition to these multiple infectious episodes, the patient developed an ectodermal phenotype including sparse and thin scalp hair, sparse and hypopigmented eyebrows, conical dysmorphic primary incisors, and other dental abnormalities (Fig. 1B, left two, and fig. S1A).

A first immunophenotyping, performed at age 10 months, identified a T cell lymphopenia, due principally to a reduction in CD4^+^ T cell numbers (see data S1 for exhaustive reporting of laboratory workups of all four patients and Table 1 for comparison of key immunological findings among these four patients). This reduction was confirmed with repeated immunophenotyping, during the course of the disease, which also established that among T cell subpopulations, there was a reduction in the number of naïve CD4^+^ and CD8^+^ T cells, an increase in the number of memory CD4^+^ T cells, and a low proportion of CD4^+^ recent thymus emigrants. In CD8^+^ T cell subpopulations, there was a marked increase in the number of effector memory (EM) cells, undetectable naïve cells, and diminished terminally differentiated EM (TEMRA) cells, while central memory (CM) cells were present in normal quantities (Table 1). Furthermore, we observed a CD19^+^ B cell lymphopenia after 2 years of age (data S1). Natural killer (NK) cells were present in normal numbers (data S1). Impaired T cell proliferation upon stimulation with phytohemagglutinin (PHA), anti-CD3, or tetanus toxin was also demonstrated as was a disparate immune response to vaccines (Table 1). Epstein-Barr virus (EBV) was detected at 5.3 log during its primary infection, concomitant with the bocavirus pneumonia (the patient EBV-seroconverted at a later time, see Table 1). Immunoglobulin levels were within the normal range (Table 1 and table S1).

**Table 1.** Highlights of the phenotype of *ITPR3* patients (for exhaustive immunological data, see data S1). Ab, antibody; VZV, varicella-zoster virus; ASD, autism spectrum disorder.

	Patient 1 (2 years 2 months)	Patient 2 (7 years 10 months)	Patient 3 (19 years 5 months)	Patient 4 (10 weeks)
**Total lymphocytes/μl**	6859 (*2790–6350*)	596 (*2280–3820*)		2500 *(3680–7340)*
**T cells**				
CD3^+^ %	87 *(53.88–72.87)*	53.76 *(60.05–74.8)*	59 *(57.1–73.43)*	53 *(54.28–71.67)*
CD3^+^/μl	5967 *(1794–4247)*	371 *(1424–2664)*	675 (*1325–2276)*	1325 *(2179–4424)*
CD4^+^/μl	754 (*902–2253)*	172 (*686–1358)*	412 (*531–1110)*	275 (*1461–3018)*
CD8^+^ %	75 (*19–39.51)*	16.84 *(19.68–34.06)*	21 *(21.01–33.94)*	37 *(14.08–24.7)*
CD8^+^/μl	5144 *(580–1735)*	160 (*518–1125)*	240 (*480–112)*	925 *(556–1687)*
**B cells**				
CD19^+^ %	9 (*13.23–26.39)*	11 *(10.21–20.12)*	21 *(9.19–19.48)*	14 *(17.34–36.03)*
CD19^+^/μl	617 *(531–1521)*	63 *(280–623)*	240 *(216–536)*	350 *(734–2275)*
**NK cells**				
CD56^+^CD16^+^ %	3 *(7.21–20.90)*	21.6 *(9–22.24)*	20 *(10.01–26.98)*	31 *(5.89–14.85)*
CD56^+^CD16^+^/μl	206 *(155–565)*	129 (*258–727)*	229 (*246–792)*	775 (*290–780)*
**% in CD4** ^**+**^ ** T cells**				
Naïve (CD45RA^+^/RO^−^)	12 *(61.8–85)*		17 *(53.3–74)*	31 *(81–91.5)*
Memory (CD45RA^−^/RO^+^)	88 *(14.8–37.2)*		47 *(22.1–36.6)*	55 *(5.1–11.4)*
Naïve (CD45RA^+^CCR7^+^)		13.8 *(45.56–75.28)*		
CM (CD45RA^−^CCR7^+^)		48.1 *(22.06–46.46)*		
EM (CD45RA^−^CCR7^−^)		37.2 *(2.08–8.78)*		
TEMRA (CD45RA^+^CCR7^−^)		0.9 *(0–1.06)*		
**% in CD8** ^**+**^ ** T cells**				
Naïve (CD45RA^+^CCR7^+^)	0 *(36.8–83.16)*	14.1 *(41.58–77.9)*		
CM (CD45RA^−^CCR7^+^)	9 *(5.18–31.66)*	23.2 *(12.08–30.54)*		
EM (CD45RA^−^CCR7^−^)	88 *(0.7–11.22)*	46 *(1.58–13.18)*		
TEMRA (CD45RA^+^CCR7^−^)	3 *(0.84–33.2)*	16.8 *(1.7–24.62)*		
**T cell proliferation**				
PHA	Decreased	Decreased	Normal	
Anti-CD3 (OKT3)	Decreased	Decreased		Low normal
VZV	Negative			
Tetanus toxoid	Negative		Decreased	
Candida			Normal	
**Postvaccine serology**				
Pneumococcus/Tetanus	Low	Low	Low	
Mumps	Positive			
IgG rubella	Positive	Positive		
IgG measles	Intermediate	Positive		
**Postinfection serology**				
Hepatitis A/HSV IgG	Positive			
HBV HBs Ag, HBc Ab/HCV/HIV/HTLV	Negative	Negative		
HBV HBs Ab	Positive	Positive		
EBV VCA IgG	Delayed	Positive		
EBV EBNA IgG	Positive	Positive		
CMV IgG	Positive	Positive		Positive
VZV IgG	Positive	Positive		
Immunoglobulins*				
IgG (g/liter)	6.3 *(4.42–11.39)*	11.9 *(6.35–12.84)*	Normal^†^	5.9 *(1.77–5.83)*
IgA (g/liter)	1.45 *(0.21–1.15)*	1.25 *(0.32–1.91)*		0.76 *(0.14–1.5)*
IgM (g/liter)	0.34 *(0.43–1.84)*	0.119 *(0.44–1.9)*		0.52 (*0.41–1.64)*
**Antinuclear antibodies**		Negative		Negative
**Growth failure**	−	+	−	+
**Neurological abnormalities**				
Intellectual disability	−	−	−	+
Motor delay	+	+	+	+
Speech delay	+	+	+	+
Abnormal behavior	−	−	+ (ASD)	+
Seizures	−	−	−	−
Hypertonia/hypotonia	−	+	+	−
Hearing loss	−	+	−	+
Brain abnormalities	^‡^	−	−	−
Taste defect	−	−	−	Too young to detect
**Congenital malformations**				
Cardiac	−	−	+	−
Urogenital/kidney	−	−	−	−
Eye	−	−	−	−
Craniofacial dysmorphism	−	+	−	−
**Skeletal abnormalities**				
Joint hypermobility	−	−	−	
Pectus deformity	+	−	+	−
Limbs	−	−	+ (Knee and elbow contractures, hammertoes)	−
Thorax	−	−	+ (Kyphoscoliosis)	−
Palate	−	−	−	−
**Ectodermal dysplasia**				
Hair abnormality	+	+	+ (Hypopigmentation)	+
Teeth abnormality	+	+	−	+

*Immunoglobulin levels are those within days/weeks of the patient age as mentioned at the top of each column.

†Exact levels not documented in patient file.

‡Not determined (ND).

In summary, this index case presented with severe combined immunodeficiency (SCID) of unknown genetic etiology, with profound CD4 and CD8 naïve T cell lymphopenia alongside an ectodermal phenotype. At 2 years and 8 months of age, he underwent a HCT with bone marrow donated by his healthy HLA-identical brother. The graft and its follow-up were uneventful, and since then, the patient has not shown any notable medical events. His height remains slightly below normal (between −1SD and −1.5SD).

Patient 2 is a 9-year-old boy born to healthy nonconsanguineous parents of Ashkenazi Jewish descent from Israel (Fig. 1A, family 2). He has three healthy sisters. From the age of 9 months onward, he presented with recurrent bacterial and viral infections. These included recurrent pneumonia and pneumococcal bacteremia at 9 to 14 months of age, postvaccination varicella at age of 14 months, pneumococcal meningitis at age of 15 to 16 months, osteomyelitis at age of 22 months, recurrent mastoiditis at the age of 26 months, and again pneumonia and another episode of pneumococcal meningitis at 3 years of age. The patient further presented ectodermal defects including sparse and thin light-colored scalp hair and eyebrows, dry skin, white-toned nail beds, and cone-shaped mandibular primary incisors (Fig. 1B, middle two, and fig. S1B). He also had developmental delays and short stature with his height measurements lying outside −2SD. The patient was first treated symptomatically and then with antibiotic prophylaxis and intravenous immunoglobulin (IVIG), but given his continued clinical deterioration, he underwent HCT at age of 8 years, the donor being one of his HLA-matched sisters. His posttransplant recovery was complicated by mild skin chronic graft-versus-host disease, which responded well to treatment with ruxolitinib.

Pretransplantation blood immunophenotyping showed also in this case CD4^+^ and CD8^+^ T cell lymphopenia and an inversion in the CD4^+^/CD8^+^ T cell ratio (Table 1 and table S1). Within the CD4 and CD8 subpopulations, there were fewer naïve cells but augmented EM and TEMRA cell numbers, and CD4^+^ CM T cells were in near normal quantities (Table 1). B and NK cell numbers were both on the lower end of normal values; this was more pronounced for B cells. Immunoglobulins (Ig) showed low IgM levels, undetectable levels of IgE, and normal levels of IgG and IgA. Postvaccinal antibody titers were normal except for a poor response to pneumococcal polysaccharide vaccine. Last, in vitro T cell proliferation in response to anti-CD3 and PHA was reduced (Table 1). A panel cytokine screening—interleukin-1β (IL-1β), IL-2, IL-2R, IL-5, IL-6, IL-8, IL-10, tumor necrosis factor–α (TNFα), TNFβ, interferon-α (IFN-α), IFN-γ—revealed above normal levels for IL-1β (0.8 pg/ml; normal range, <0.2 pg/ml), IL-2R (4681 pg/ml; normal range, 688 to 1872 pg/ml), TNFα (20.9 pg/ml; normal range, 4.9 to 9.7 pg/ml), and IFN-γ (5.3 pg/ml; normal range, 0.5 to 2.7 pg/ml). Antinuclear antibodies (including anti-Ro/SS-A, anti-La/SS-B, anti-Sm, and anti-RNP) were negative (Table 1).

Patient 3 is a 19-year-old US white male (Fig. 1A, family 3) with a complex history of multisystem abnormalities born to healthy nonconsanguineous parents of European descent after an uncomplicated pregnancy and vaginal delivery with no neonatal complications. His only sibling, a 13-year-old brother, is healthy. Starting in his early childhood, he suffered from recurrent infections including multiple episodes of pneumonia, sinusitis, otitis media, and mastoiditis. He also missed numerous developmental and social milestones including delayed language and motor development and developed several physical deformities, e.g., pectus carinatum, pes cavus, bilateral cavovarus feet deformity, as well as a number of chronic functional disabilities, whether respiratory (asthma), gastrointestinal (constipation), bone (osteoporosis), or neuropsychiatric (autism spectrum disorder). Last, demyelinating polyneuropathy, as corroborated by neurologic examination and imaging, was consistent with the diagnosis of Charcot-Marie-Tooth disease.

Immunologically (and unlike the two previous patients, and like the next patient, this was not his primary medical concern), starting in early childhood, he developed recurrent pulmonary, sinus, and ear infections. He also had leukopenia. Treatment with monthly IVIG resulted in improved health. Poor weight gain necessitated a temporary gastrostomy tube placement. His hair was very light in color (picture unavailable). Routine immunological workup revealed persistent lymphopenia, with decreased absolute numbers of total T cells as well as of CD4^+^ and CD8^+^ T cells. T cell response to PHA and *Candida albicans* was normal, but proliferative response to tetanus toxoid was decreased (Table 1). In summary, he was found to have a primary immunodeficiency displaying a CD4^+^ and CD8^+^ T cell lymphopenia within the broader context of a multisystemic disease.

Patient 4 is a North American 36-month-old white male born at term following an unremarkable pregnancy and cesarean section due to failure to progress (Fig. 1A, family 4). The parents are healthy, nonconsanguineous, and of European and Asian descent. His disease was first detected through the state’s newborn screening program for SCID in the United States.

Initial immunophenotyping revealed profound CD4 T cell lymphopenia and moderate CD8 T cell lymphopenia, with a low count of naïve T cells and an increase in memory T cells. B cells were diminished. Igs were at normal levels, whereas lymphocyte mitogen stimulation demonstrated a low-normal response to PHA, concanavalin A and pokeweed mitogen (Table 1 and table S1). His main entry into pathology was at 12 months of age, when he presented with pancytopenia, conical primary incisors (Fig. 1B, right two, and fig. S1C), hepatosplenomegaly, and generalized lymphadenopathy. Lymph node and bone marrow biopsies revealed atypical lymphohistiocytic proliferation, and findings were presumed to represent Rosai-Dorfman disease at the time (fig. S2). Pancytopenia responded to a short course of steroids. No brain or spinal cord involvement was detected. The child was hospitalized at 13 months of age with respiratory distress secondary to pneumonia. Global developmental delay (GDD) was also noted. The clinical course was further complicated by several events, including unilateral facial palsy and bilateral mastoiditis treated with bilateral mastoidectomy, amoxicillin/clavulanic acid, and systemic steroids. Pathologic findings showed reactive benign lymphohistiocytic infiltrates causing effacement of marrow hematopoietic cells leading to hypo-productive bone marrow failure (fig. S2). After stopping steroids, he again presented pancytopenia, lymphadenopathy, and hepatosplenomegaly with lymph node biopsy showing abnormal infiltration by benign histiocytes at 17 months of age, and he was started on treatment with clofarabine (no response after two courses), followed by vinblastine and systemic steroids. At 23 months of age, a bone marrow biopsy demonstrated a persistent lymphohistiocytic process (fig. S2) when the child was placed on trametinib for 2 months to help wean off steroids. Last, all medications except steroids were stopped. In addition, he had CMV reactivation and GDD was still present (growth faltering since 4 months of age, from 0SD to −1.75SD), while the remainder of the clinical exam was unremarkable. Antinuclear antibodies (including anti-Ro/SS-A, anti-La/SS-B, anti-Sm, and anti-RNP) were negative. Within the tested range of anti-cytokine antibodies—anti–IFN-α, anti–IFN-β, anti–IFN-ω, anti–IL-17, anti–IL-17F, anti–IL-22—only anti–IL-22 autoantibodies were clearly positive (fig. S3) (*13*). In sum, this patient entered the disease phase through what could be qualified as a hemophagocytic lymphohistiocytosis (HLH)–like (as we do not have all the required information to formally classify the syndrome as bona fide HLH) syndrome (*14*). He recently underwent a T cell–depleted haploidentical HCT after a treosulfan-based conditioning and has engrafted with full donor chimerism.

### Identification of an *ITPR3*-dominant variant as the culprit

Whole-exome sequencing (WES) was performed in all four patients and their parents (and in the case of family 2 in a healthy sibling) to identify the causal gene (Fig. 1A). In all four independent families, the same unique missense variant in the *ITPR3* gene (NM_002224.3:c.7570C>T, p.Arg2524Cys) segregated with the disease in a de novo inheritance model, i.e., heterozygous in patients while absent [wild type (WT)] in parents and all other tested family members. The variant was further confirmed by targeted Sanger sequencing in families 1 and 2 (fig. S4). The identified variant was absent in the Genome Aggregation Database and our in-house (Strasbourg) database (~1000 WES of mainly inborn errors of immunity). In CENTOGENE’s Biodata Bank of 135,905 individuals with exome/genome sequence, this variant was seen only twice, in patient 2 and in an individual with an identical phenotype to patients reported in this work, which is not further described here due to lack of documented parental consent. No other potentially causal protein-coding variant(s) passing our filtering criteria (see Materials and Methods) was identified in either of the families, including in any alternative inheritance mode [X-linked or autosomal recessive—homozygosity or compound heterozygosity; see table S1 for all identified variants and Discussion with respect to recent literature on the subject (*12*)]. In silico analyses predicted the variant to be deleterious (score 0, scale-invariant feature transform), probably damaging [score, 0.999; Polyphen2, Polymorphism Phenotyping v2 (PolyPhen)], and damaging (score, 1; The Mutation Taster tool) (Table 2).

**Table 2.** *ITPR3* candidate variant position and predicted impact. Positions refer to GRCh37 (hg19) reference genome. SIFT, scale-invariant feature transform; PolyPhen, Polyphen2, Polymorphism Phenotyping v2; CADD, Combined Annotation Dependent Depletion Phred scaled. GERP++ Genome Evolutionary Rate Profiling ++: the larger the score, the more conserved the site. PhastCons30way_mammalian: conservation score based on the multiple alignments of 30 mammalian genomes, the larger the score, the more conserved the site.

Position	*chr6:33660616*
Gene symbol	*ITPR3*
ref	C
alt	T
dbSNP	NA
Consequence	Missense variant
HGVSc: Nucleotide change	NM_002224.3:c.7570C>T
HGVSp: Protein change	NP_002215.2:p,Arg2524Cys
SIFT (score)	Deleterious (0)
PolyPhen (score)	Probably damaging (1)
CADD (phred score)	32
GERP++_RS	4.53
MutationTaster	Disease causing
phastCons30way_mammalian	0.95

### ITPR3 mRNA and protein levels in patients and CRISPR-Cas9–edited Jurkat cells

**Fig. 2.**
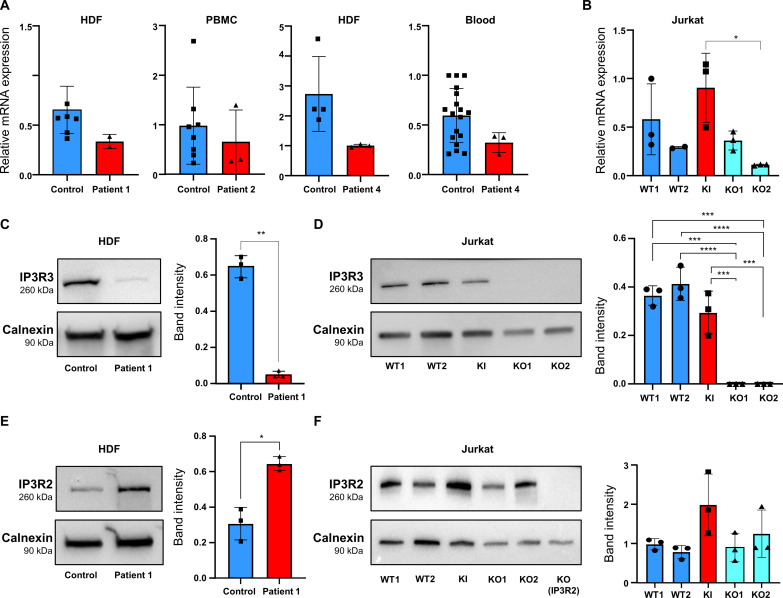
Effects of the *ITPR3* variant on *ITPR3* mRNA and IP3R2 and IP3R3 protein expression. (**A**) *ITPR3* mRNA expression in patient 1’s HDFs (*n* = 2 independent experiments), patient 2’s PBMCs (*n* = 3), patient 4’s HDFs (*n* = 3), and blood (*n* = 3) versus those from various controls and (**B**) in CRISPR-Cas9–edited Jurkat cells (*n* = 3, KI = IP3R3 R2524C knock-in, KO = IP3R3 knock-out) as measured by reverse transcription quantitative polymerase chain reaction (RT-qPCR), normalized to housekeeping genes *GAPDH* and β*-actin*. (**C** and **D**) Representative Western blot of IP3R3 (top) and calnexin (bottom) in (C) patient 1’s HDFs versus age-matched control HDFs and (D) in five CRISPR-Cas9–edited Jurkat cells. Right [(C) and (D)]: Relative IP3R3 expression quantification by Western blot band intensity normalized to calnexin (*n* = 3 independent experiments each). (**E** and **F**) Representative Western blot of IP3R2 (top) and calnexin (bottom) in (E) patient 1’s HDFs compared to control HDFs and (F) in the above-described five CRISPR-Cas9–edited Jurkat cells as well as in a negative control (IP3R2 CRISPR-Cas9 KO in Jurkat cells). Right [(E) and (F)]: Relative IP3R2 expression quantification by Western blot band intensity normalized to calnexin [*n* = 3 for both (E) and (F)]. All values are represented as mean ± SD. Statistics: (A), (C), and (E): *T* test; (B), (D), and (F): One-way analysis of variance (ANOVA) [*P < 0.05, **P < 0.01, ***P < 0.001, ****P <0.0001, and *P* > 0.05 (not shown)]. Data values are provided in Table S3.

Human dermal fibroblasts (HDFs) of patients 1 and 4, peripheral blood mononuclear cells (PBMCs) of patient 2 (before HCT), and whole blood of patient 4 (before HCT) were used to assess the expression of the *ITPR3* gene and the IP3R3 protein (for logistical/practical reasons, we did not have access to any biological material from patient 3; so all experiments throughout the manuscript were performed on primary or derived cells from patients 1, 2, and 4, all before HCT). In all patients, WT and variant alleles of *ITPR3* mRNA were found to be nearly equally expressed (no statistically significant difference) as assessed by RNA sequencing (RNA-seq) (fig. S5). *ITPR3* mRNA was found to be equally expressed in all biological samples (HDFs, PBMCs, and whole blood) of patients compared to healthy individuals (with a tendency of lower expression in patient cells versus controls yet not reaching statistical significance) (Fig. 2A). However, IP3R3 protein expression was significantly down-regulated in patient 1 HDFs compared to age-matched control HDFs, as shown by Western blotting (Fig. 2C).

To analyze the impact on mRNA and protein levels of this single variant of *ITPR3* [and not any other variant(s) in any other mode of inheritance including autosomal recessive with compound heterozygous zygosity; cf. discussion of recent literature in Discussion section], we introduced the *ITPR3* c.7570C>T variant in the Jurkat T cell line using CRISPR-Cas9. Among the five analyzed cell lines, two remained WT (WT1 and WT2), one carried the heterozygous c.7570C>T knock-in (KI) variant, and two became knockout (KO) by deletion of 1 (KO1) or insertion of 2 (KO2) nucleotides, as verified by targeted Sanger sequencing (fig. S7A). RNA-seq read alignments at *ITPR3* variant coordinates (chr6:33660616, c.7570C>T, p.R2524C) showed again that KI Jurkat cells (similar to patient cells) equally expressed WT and variant allele transcripts (fig. S5). Consistent with what is observed in patient cells, no significant difference in total *ITPR3* mRNA levels was observed in KI versus WT Jurkat cells, whereas a significant decrease of *ITPR3* mRNA expression was detected in KI versus WT cells by reverse transcription quantitative polymerase chain reaction (RT-qPCR) (Fig. 2B) and in KO versus WT by RNA-seq (fig. S7B). Western blot analysis of CRISPR-Cas9–edited Jurkat cell lines showed a slight decrease in IP3R3 levels in KI versus WT1 (Fig. 2D). Using band intensity quantification, we estimated the remaining expression of IP3R3 to 80 and 70% of WT1 and WT2, respectively (Fig. 2D, right). IP3R3 was absent in both KO (KO1 and KO2) Jurkat cells (Fig. 2D). Collectively, these results indicated that the *ITPR3* variant identified here did not significantly alter *ITPR3* mRNA expression but reduced IP3R3 protein levels.

We further assessed the impact of the IP3R3 R2524C variant on the expression of the tetramer component IP3R2 by Western blot. Patient 1 HDFs showed an around twofold increase of IP3R2 expression compared to control (Fig. 2E), while in Jurkat IP3R3 KI and KO cells, IP3R2 was not significantly different as compared to WT (Fig. 2F).

### A dominant-negative mechanism of calcium flux defects in patients and engineered Jurkat cells

**Fig. 3.**
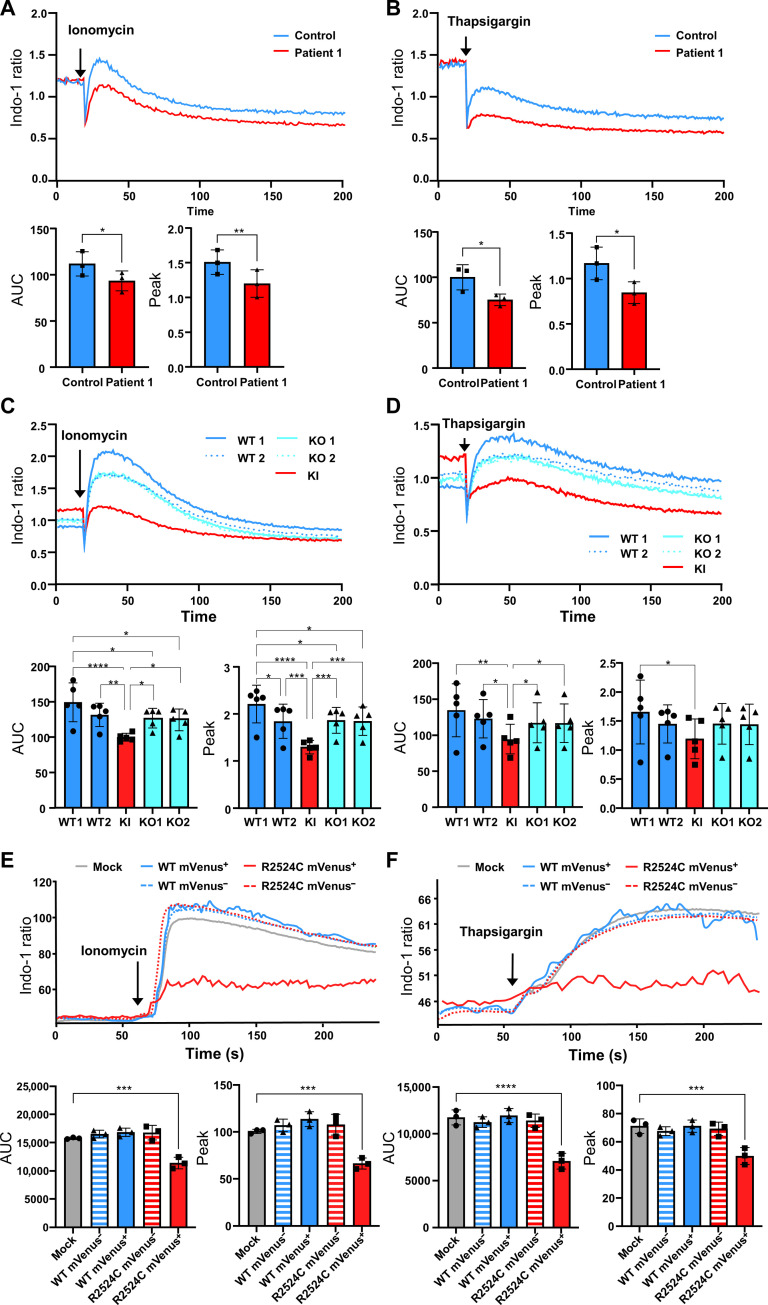
Consequences of *IP3R3* R2524C variant on calcium flux. (**A** to **D**) Calcium flux kinetics of patient 1’s HDFs (*n* = 3) and Jurkat cells (*n* = 5) measured by the Ca^2+^ indicator Indo-1 fluorescence ratio after stimulation (arrow) with 1 μM ionomycin [(A) and (C)] or 1 μM thapsigargin [(B) and (D)] in the presence of 5 mM EGTA over 200 time points with 650-ms intervals (mean of *n* = 3 independent experiments). Bottom left represents mean AUC, and bottom right represents mean peaks. (**E** and **F**) Representative calcium flux kinetics of Jurkat cells (from *n* = 6 replicates) transfected with the indicated *ITPR3*-expressing plasmids as measured by the Ca^2+^ indicator Indo-1 fluorescence ratio after stimulation (arrow) with 1 μM ionomycin (E) or 1 μM thapsigargin (F). All bar graph values are represented as mean ± SD. Statistics: (A) and (B): *T* test; (C) to (F): One-way ANOVA [*P < 0.05, **P < 0.01, ***P < 0.001, ****P < 0.0001, and *P* > 0.05 (not shown)]. Data values are provided in table S3.

HDFs from patient 1 were loaded with the ratiometric calcium probe Indo-1 and stimulated with 1 μM ionomycin in the presence of 5 mM EGTA. A decreased signal in the calcium flux kinetics compared to age-matched control HDFs was observed (Fig. 3A, top), with both an area under the curve (AUC) and a maximum peak being significantly lower than age-matched control HDFs (Fig. 3A, bottom). Stimulation with 1 μM thapsigargin, a sarcoplasmic/ER Ca^2+^–adenosine triphosphatase inhibitor, in the presence of 5 mM EGTA, allowed the investigation of ER Ca^2+^ levels in HDFs. Similar to the response with ionomycin, patient 1’s HDFs showed a decreased response in calcium kinetics when stimulated with thapsigargin compared to age-matched control HDFs (Fig. 3B, top), with both an AUC and a maximum peak significantly lower compared to age-matched controls (Fig. 3B, bottom). A similar defect in calcium mobilization was observed in patient 4’s T cells (fig. S6). Hence, these results demonstrated that patient cells display a defective calcium flux function and a decreased stored Ca^2+^ level in the ER.

The calcium kinetics were again measured in Jurkat KI cells by loading the cells with the ratiometric calcium probe Indo-1 and upon stimulation with 1 μM ionomycin in the presence of 5 mM EGTA. Heterozygous KI Jurkat cells showed a decreased signal in the calcium flux kinetics compared to all other Jurkat cell lines (WT1, WT2, KO1, and KO2) (Fig. 3C, top), with both an AUC and a maximum peak significantly lower in the KI cells than in all the other Jurkat cell lines (WT1, WT2, KO1, and KO2) (Fig. 3C, bottom). Stimulation with 1 μM thapsigargin in the presence of 5 mM EGTA also resulted in a decreased level of signal in the calcium flux kinetics of heterozygous KI Jurkat cells compared to all other Jurkat cells (WT1, WT2, KO1, and KO2) (Fig. 3D, top), with a significantly lower AUC in the KI cells compared to all other cell lines (Fig. 3D, bottom) and a lower maximum peak when compared to all other CRISPR-Cas9–edited Jurkat cells, although significance was reached only when compared to WT1 (Fig. 3D, bottom right). The calcium flux kinetics, AUC, and maximum peak of KO1 and KO2 were very similar to those of WT2 and, to some extent, to those of WT1 (Fig. 3, C and D). Thus, the introduction of the heterozygous missense c.7570C>T variant alone in Jurkat cells entirely recapitulated the abnormal calcium flux phenotype observed in patient 1’s HDFs. Also, the similarity between the two KO results with WT2 and, to some extent, WT1, combined with the significant differences between the two KO and the KI, suggests that the *ITPR3* heterozygous c.7570C>T variant specifically impairs calcium flux function and cannot be replicated by a KO of the gene, suggesting a dominant-negative effect for the variant*.* To experimentally confirm this hypothesis, we transiently transfected Jurkat cells with plasmids encoding either the WT or the R2524C variant of IP3R3 respectively fused to mVenus at their N terminus. Two days after transfection, both the transfected cells and a mock control were loaded with Indo-1 and stimulated either with 1 μM ionomycin or 1 μM thapsigargin to investigate the effect of the overexpression of either the WT or the R2524C variant on the calcium response of Jurkat cells. As the mVenus negative (mVenus^−^) populations do not express the vector-derived *ITPR3* transcripts, they can be considered as negative controls and showed indeed almost identical calcium flux kinetics (AUC and peak) to mock cells (Fig. 3, E and F). Similarly, overexpression of the WT IP3R3 did not alter the calcium response, no difference could be detected between the mVenus positive (mVenus^+^) population expressing WT IP3R3 and negative controls (mVenus^−^ and mock cells). However, a significant lower AUC and diminished peak were observed for the mVenus^+^ population of Jurkat cells transfected with the plasmid encoding the IP3R3 R2524C variant (Fig. 3, E and F), demonstrating a dominant-negative effect of the patient’s variant.

### Exquisite sensitivity of IP3R3’s Arginine 2524 to change

**Fig. 4.**
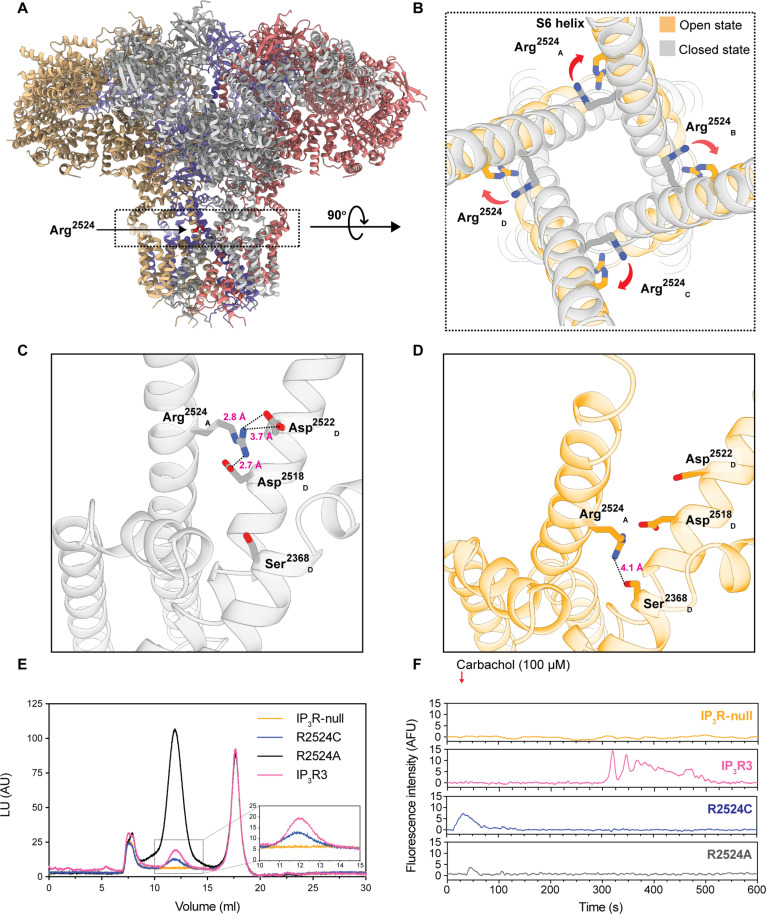
Structure function assessment of *IP3R3 R2524* variants. (**A**) Cartoon representation of the side view of tetrameric human IP3R3. R2524 is located in the dashed box that is shown in (B). (**B**) Superposition of closed and open states of IP3R3 (residues 2488 to 2537) highlighting the movement of R2524 during transition from the closed, resting state (gray) to an open, activated state (orange). (**C** and **D**) Representation showing interprotomer interactions formed by R2524 in the resting state (C) and in the activated state (D). (**E**) Fluorescence detection size exclusion chromatography profile of HEK293T-IP3R-null cells and cells expressing WT-IP3R3, R2524C, R2524A, or R2524E. Inset magnifies the ~12-ml peak for cells expressing WT-IP3R3, R2524C, or untransduced cells. The ~8-ml peak corresponds to the void volume of the column, which is present in all samples, The ~12-ml peak corresponds to folded, tetrameric channels and is absent from the untransduced cells. The ~16-ml peak corresponds to an endogenous protein that we can detect in all samples. (**F**) Representative Cal-520-AM fluorescence traces recorded from a cell expressing WT-IP3R3, R2524C, R2524A, and R2524E variants, respectively, in an IP3R-null background following stimulation by carbachol. LU, luminescence units; AU, arbitrary units; AFU, arbitrary fluorescence units.

Human IP3R3 is an ER membrane–resident tetrameric cation channel. Each protomer of human IP3R3 consists of 2671 residues and is composed of a large cytoplasmic domain at the N terminus, followed by a juxtamembrane domain, a transmembrane domain (TMD), and a C-terminal domain. The ion conduction pathway of IP3R3 is formed by the S5, S6, and reentrant pore helices within each subunit’s TMD (Fig. 4) (*5*).

R2524 is located on the pore-lining S6 helix of the TMD of IP3R3, where it forms state-dependent interactions with residues of the adjacent protomer and influences the electrostatic profile of the pore (Fig. 4, A and B) (*5*, *15*). In states with a closed pore, R2524 faces toward the pore, where it forms salt bridges with D2518 and D2522 of the neighboring protomer (Fig. 4C). Upon activation, R2524 rotates away from the pore and instead interacts with S2368 of the same protomer (Fig. 4D). Because of its central location in the pore and its role in interprotomer salt bridges, we hypothesize that R2524 is critical for channel function. To assess the effect of variant(s) at R2524 on channel folding, we generated baculoviruses carrying WT or variant *ITPR3* fused to an N-terminal fluorophore gene sequence. Human embryonic kidney (HEK) 293T cells lacking endogenous *ITPR1*, *ITPR2*, and *ITPR3* (HEK293T-IP_3_R-null) were transduced with the baculovirus, and then proteins were solubilized in detergent and subjected to fluorescence detection size exclusion chromatography (*5*, *16*). Consistent with previous results, WT channels eluted as a monodisperse peak with a retention volume of ~12 ml, corresponding to a tetrameric channel (Fig. 4E) (*5*). While the expression levels varied for the R2524A, R2524C, and R2524E variants, they too eluted at ~12 ml, indicating that substitutions to R2524 do not disrupt the tetrameric architecture. We next examined the effect of these variants on cytosolic Ca^2+^ oscillations in cells lacking the three endogenous *ITPR* genes. By monitoring Cal-520-AM fluorescence, we found that cells expressing WT IP3R3 yielded robust cytosolic Ca^2+^ oscillations upon stimulation with saturating (100 μM) carbachol concentrations (Fig. 4F). In contrast, no oscillations were observed in cells expressing the R2524 variants or in the untransduced control cells. Thus, even when expressed by a strong promoter, channels that had variants at R2524 were unable to mediate Ca^2+^ oscillations.

### A mitochondrial RNA-seq and proteomic signature in patients

**Fig. 5.**
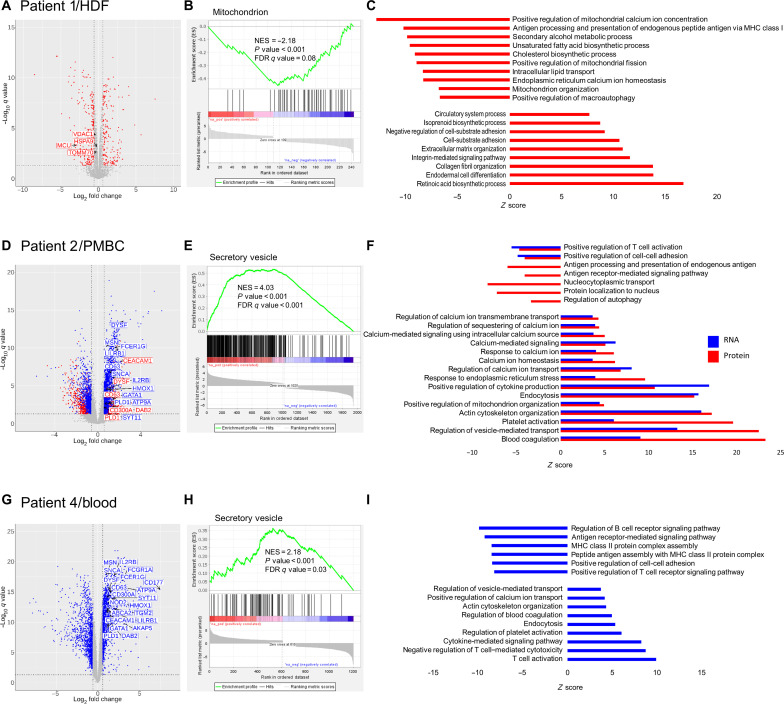
Proteomics and RNA-seq analyses of *ITPR3* patients. (**A**) Volcano plot representing the differentially expressed proteins in dermal fibroblasts of patient 1 compared to four controls. The red dots represent the proteins that are differentially expressed with a corrected *P* value < 0.05 and a minimal fold change of 1.5. Labels correspond to proteins of the MAMs family that are all down-regulated. (**B**) GSEA plot showing negative enrichment of proteins of the mitochondrial cellular compartment. (**C**) Positive and negative enrichment of Gene Ontology (GO) cellular processes in fibroblasts of patient 1. (**D**) Volcano plot representing the differentially expressed genes and proteins in PBMCs of patient 2 compared to six controls. The dots represent the proteins (red) and the transcripts (blue) that are differentially expressed with a corrected *P* value < 0.05 and a minimal fold change of 1.5. Labels correspond to up-regulated genes and up-regulated proteins of the GO category “regulation of vesicle-mediated transport.” (**E**) GSEA plot showing positive enrichment of proteins of the secretory vesicle pathways. NES, normalized enrichment score. (**F**) Positive and negative enrichment of GO cellular processes in patient 2. (**G**) Volcano plot representing the differentially expressed genes in whole blood of patient 4 compared to three age-matched controls. The blue dots represent genes that are differentially expressed with a corrected *P* value < 0.05 and a minimal fold change of 2. Labels correspond to up-regulated genes of the GO category regulation of vesicle-mediated transport. (**H**) GSEA plot showing positive enrichment of genes of the secretory vesicle pathways. (**I**) Positive and negative enrichment of GO cellular processes in whole blood of patient 4.

IP3R3 is a calcium channel that is highly expressed at the ER-mitochondria interface, where it provides Ca^2+^ for transport into the mitochondria and the regulation of energy production (*17*, *18*). ER-mitochondria contacts and the transport of Ca^2+^ into the mitochondria occur via mitochondria-associated membranes (MAMs), which account for ~20% of the mitochondrial surface (*19*). Differential expression analysis of proteomic data from patient 1 fibroblasts showed that MAMs such as the mitochondrial Ca^2+^ uniporter or the voltage-dependent anion channel 1 located at the mitochondrial membrane were down-regulated in patient’s cells (Fig. 5A). Globally, the mitochondrial cellular compartment is shown to be negatively affected by the IP3R3 R2524C variant (Fig. 5B). Consistent with the known role of the IP3R3 pathway in negative regulation of autophagy by the control of mitochondrial Ca^2+^ levels (*20*), the positive regulation of macroautophagy and mitochondrial-related pathways was also shown to be down-regulated in patient 1 (Fig. 5C). In PBMCs of patient 2, differential expression analysis of proteomic and RNA data (Fig. 5D) showed a negatively affected regulation of T cell activation and antigen processing and a positively affected calcium homeostasis, regulation of mitochondrial organization, cytokine production, and vesicle-mediated transport. Gene set enrichment analysis (GSEA) showed a positive enrichment of protein secretory vesicle pathways (Fig. 5, E and F). RNA-seq on whole blood from patient 4 showed similar results (Fig. 5, G to I). Hence, IP3R3 R2524C seems to increase the vesicular trafficking [see labels on volcano plots in Fig. 5 (D and G)] (*21*).

RNA-seq analysis on the CRISPR-Cas9 *ITPR3* KI versus WT cells showed a down-regulation of the transforming growth factor–β signaling that is known to be involved in blocking Ca^2+^ influx and thereby T cell activation (*22*) and positive regulation of leukocyte activation and immune response. The up-regulated pathways were mostly related to cytoskeleton and intracellular organization and transport, in line with what was observed in blood (fig. S8).

### Altered subcellular IP3R3 localization in patient fibroblasts

**Fig. 6.**
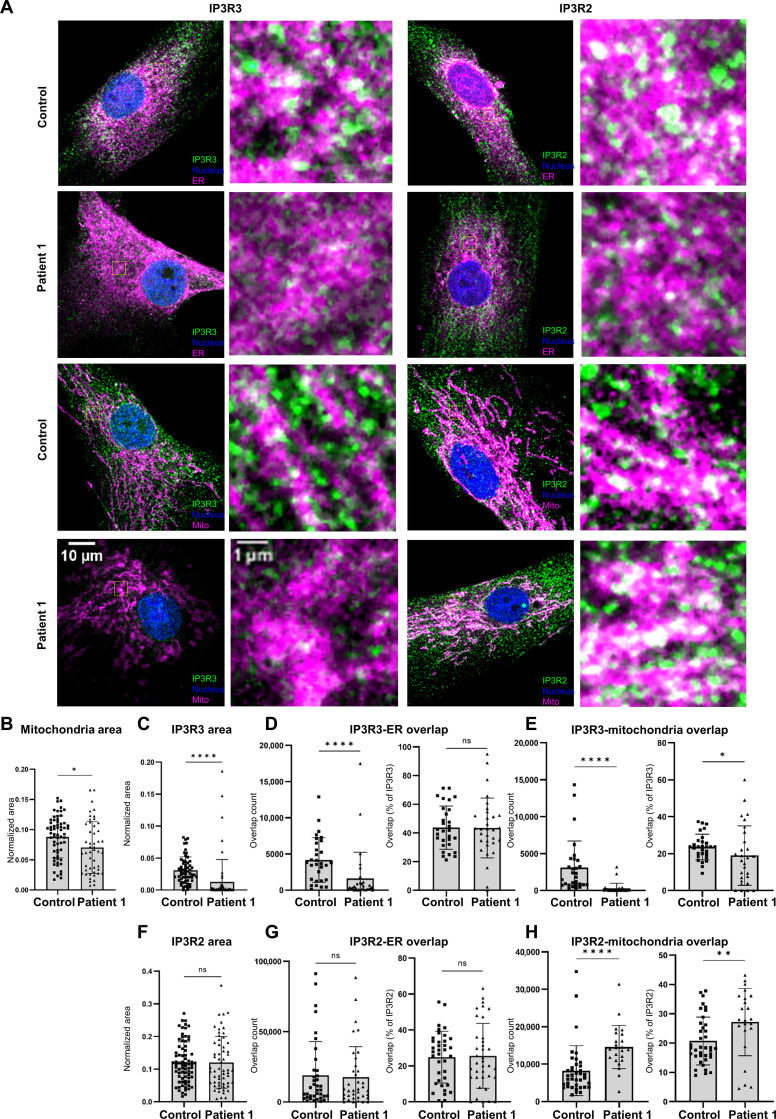
IP3R3 localization in patient 1 HDFs by immunofluorescence and confocal microscopy. (**A**) Confocal microscopy images of patient 1 and age-matched control HDFs labeled against IP3R3 (left two columns) (green), IP3R2 (right two columns) (green), ER (top, magenta), or mitochondria (bottom, magenta). Each condition is represented as a whole cell image and a ×40 magnification of regions highlighted by yellow squares. All conditions were also labeled for nuclei (blue). Colocalizing regions appear in white. (**B**) Mitochondrial area normalized to the total cell area of patient 1 and age-matched control HDFs (*n* = 50 and *n* = 64, respectively). (**C**) IP3R3 area normalized to the total cell area in patient 1 and age-matched control HDFs (*n* = 59 and *n* = 59, respectively). (**D**) IP3R3 and ER area overlaps represented as total overlap count (left) and as percentage of whole cell IP3R3 area (right) (patient *n* = 31 and control *n* = 30). (**E**) IP3R3 and mitochondrial overlaps represented as the total overlap count (left) and as percentage of whole cell IP3R3 area (right) (patient *n* = 28 and control *n* = 29). (**F**) IP3R2 area normalized to the total cell area in patient 1 and age-matched control HDFs (*n* = 60 and *n* = 75, respectively). (**G**) IP3R2 and ER overlaps represented as the total overlap count (left) and as the percentage of whole-cell IP3R2 area (right) (patient *n* = 37 and control *n* = 37). (**H**) IP3R2 and mitochondria overlaps represented as the total overlap count (left) and as a percentage of whole cell IP3R2 area (right) (patient *n* = 23 and control *n* = 38). All experiments were performed in triplicate. Mann-Whitney test was used. *P < 0.05, **P < 0.01, and ****P < 0.0001. ns, not significant.

Confocal microscopy experiments were performed using patient 1 HDFs and age-matched control HDFs stained with anti-IP3R3 or anti-IP3R2 antibodies in parallel to ER (anti-calnexin) or mitochondria (MitoTracker) markers (Fig. 6A). Although cell size and ER area remained unaffected between patient 1 and age-matched control HDFs (fig. S9, A and B), both mitochondria (Fig. 6, A and B) and IP3R3 area (signal coverage relative to the whole cell area; Fig. 6, A and C) were significantly decreased in the patient compared to the age-matched control. In addition, we observed a 25% reduction of IP3R3 mean fluorescence intensity (fig. S9C). In contrast, both IP3R2 signal coverage and mean intensity were similar between the two conditions (Fig. 6F and fig. S9D). We then analyzed the colocalization of IP3R3 and IP3R2 with the ER and mitochondria (by quantifying their signal overlap). For IP3R3-ER overlaps, we observed a significant decrease in IP3R3 in the ER in patient 1’s HDFs (Fig. 6D, left), which was expected since IP3R3 levels were decreased in the whole cell. When the IP3R3-ER overlaps were shown as a fraction of the whole cellular IP3R3, there was no significant difference between patient 1 and age-matched control HDFs (Fig. 6D, right), suggesting that the ER localization of IP3R3 is not altered by its reduced expression levels. Similarly, IP3R3 and mitochondria overlaps showed a significant decrease in patient 1 (Fig. 6E, left). When represented as a fraction of the whole cell IP3R3 area, the colocalization between IP3R3 and mitochondria was significantly lower in HDFs of patient 1 compared to age-matched control (Fig. 6E, right), suggesting that IP3R3 localization in mitochondria was reduced. We similarly probed the localization of IP3R2 and observed no significant difference in colocalization with calnexin-labeled ER (Fig. 6G) between patient 1 and age-matched control. However, when carefully assessing the localization of IP3R2 in mitochondria, we observed that it was enhanced (Fig. 6H), possibly compensating for the decrease in IP3R3 levels. To further analyze the interplay between the ER and mitochondria in these cells, we performed electron microscopy experiments and probed the physical proximity of these two organelles (fig. S10A). These results showed no significant difference in the overall aspect of the mitochondria or the ER between patient 1 and age-matched control. When investigating organelle contact sites (i.e., occurring in threshold of 10-nm distance), we observed no significant difference both in terms of number of contacts (fig. S10B) or in the area of contact (fig. S10C) between patient 1 and age-matched control, suggesting that the IP3R3 variants mostly altered its localization in the ER and mitochondria, without perturbing the contacts sites.

### An immune, calcium, mitochondria, and T cell deep multiomics signature in patients

**Fig. 7.**
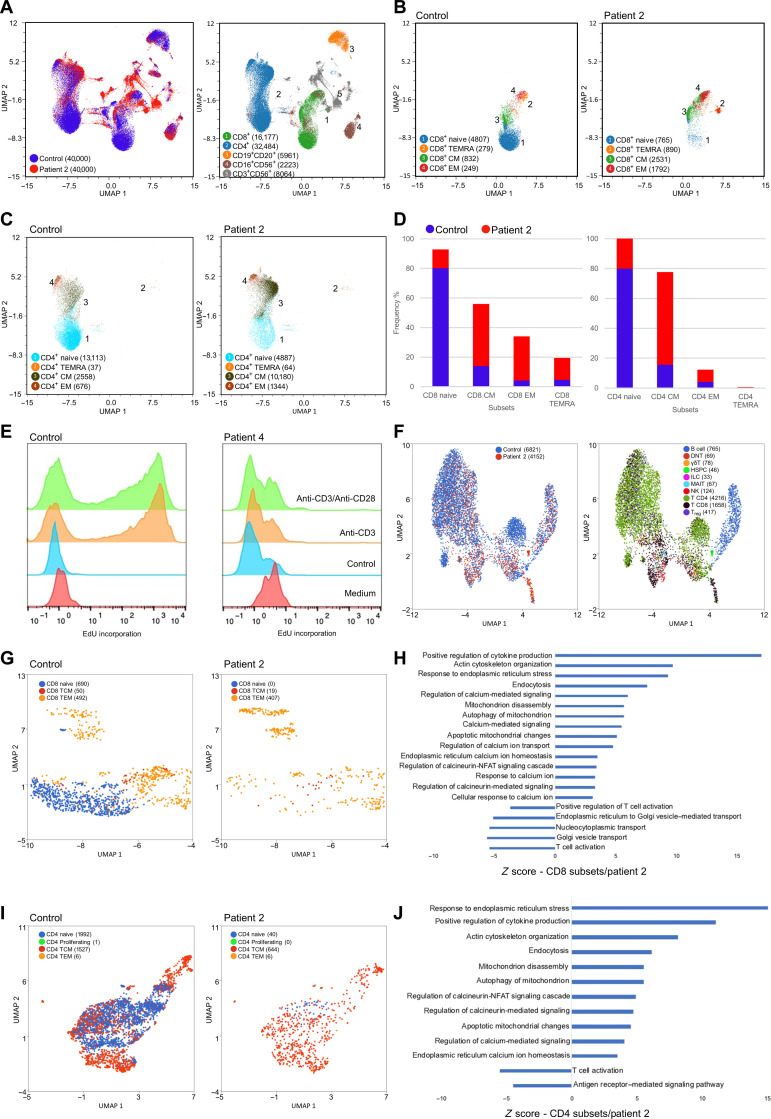
Immunophenotyping, cell proliferation, and scRNA-seq analyses. (**A**) General uniform manifold approximation and projection (UMAP) representing the major cell populations identified in the whole blood samples from patient 2 and healthy control individuals as analyzed by 40 color spectral flow cytometry (left) with a focus on T/NK cells (right). (**B**) CD8^+^ T cell subpopulations in control and patient 2 are shown on a UMAP. (**C**) CD4^+^ T cell subpopulations in control and patient 2 are shown on a UMAP. (**D**) Frequencies (bar graph) of cell repartition of naïve, EM, TEMRA, and CM cells in CD8^+^ and CD4^+^ T compartments for patient 2 and control. (**E**) Healthy control (left) and patient 4 (right) CD45^+^CD3^+^ cell proliferation upon stimulation with the indicated stimuli measured by the Click-iT EdU assay showing that patient 4 T cells are less responsive to stimulation. Data are representative of a minimum of two independent experiments. (**F**) General UMAP representing major cell populations detected in the whole blood of patient 2 and healthy control individual by scRNA-seq. (**G**) scRNA-seq–identified CD8^+^ T cell subpopulations in control and patient 2 are shown on a UMAP. (**H**) Positive and negative enrichment of GO cellular processes in scRNA-seq–identified CD8^+^ T cells of patient 2. All signaling pathways have a *q* value < 0.05. (**I**) scRNA-seq–identified CD4^+^ T cell subpopulations in control and patient 2 are shown on a UMAP. Cell numbers are indicated in parentheses. (**J**) Positive and negative enrichment of GO cellular processes in scRNA-seq–identified CD4^+^ T cells of patient 2. All signaling pathways had a *q* value < 0.05.

We performed immune phenotyping by spectral flow cytometry (Fig. 7, A to D) and single-cell RNA-seq (scRNA-seq) (Fig. 7, F to J) on peripheral blood cells of patient 2 (Table 1 and table S1). Immunophenotyping showed a decreased total lymphocyte cell count, mainly in T cells and slightly in B cells, while NK cell counts were largely normal (data S1). Within T cells, CD4 lymphopenia was more pronounced than that of CD8 lymphopenia. A relatively large increase in the number of peripheral CD4^+^CD8^+^ double-positive T cells was also observed. Among CD4^+^ T cells, there was a strong decrease in the naïve subpopulation and an increase in both CM and EM subpopulations (Fig. 7). CD4^+^ T cells also exhibited increased activation and expression levels of senescence markers CD57, CD69, CD95, and PD-1. Similarly, in CD8^+^ T cells, the naïve subpopulation was strongly decreased, the CM subpopulation hovered around the normal range, while the EM subpopulation was strongly increased. CD8^+^ T cells also exhibited increased activation and senescence markers CD57 and PD-1. Immunophenotyping of B cell subsets showed a decrease in both switched memory B cell and plasmablast numbers. Although NK cell counts did not differ from the normal range, they did exhibit an increase in the expression levels of differentiation marker CD57 and NKG2C and a decrease in the expression level of NKp30, suggesting a response to chronic infection. This more in-depth phenotype of patient 2 showed a similar profile to that of other patients, with the main features being a loss of CD4^+^ and CD8^+^ naïve T cells, an increase in both CD8^+^ and CD4^+^ EM T cell subpopulations, and subtle perturbations in other lymphoid compartments, while myeloid cells were all within the normal range, as were the Ig levels. We performed RNA-seq–based sequencing with 3′ VDJ for the T cell receptor α (TRA) and β (TRB) rearrangements of patients 2 (at a 2-week interval) and 4 and compared them to their healthy family members (father in the case of patient 2 and father and mother in the case of patient 4) and control individuals (three unrelated age-matched controls for each patient). A limited number of expanded unique CDR3 sequences were observed in both P2 and P4 consistent with restricted TRA and TRB repertoire diversity and increased clonality compared to healthy relatives and normal controls (fig. S11, A and B). For instance, in patient 2, the two largest alpha clones accounting for 25 to 35% of all T cells, and similarly for the four largest beta clones, in contrast to 3 to 4% at most in control samples. The largest TRV beta clone (TRBV4-1, 20% of all T cells) was overrepresented in flow cytometry analyses using TCRBV7S1 (TRBV4-1, TRBV4-2, and TRBV4-3) antibody (fig. S12). CDR3 length distribution was overall preserved in all samples (fig. S11C). Heatmaps representing Vα and Jα gene segments usage showed even utilization of the most distal *TRAV* and *TRAJ* genes making a defect of thymocyte survival unlikely (fig. S11D) (*23*). In total T cells, a skewed usage toward TRAV13-1, TRAV21, TRAJ42, and TRBV5-1 in patient P4 and TRAV26-2 and TRAJ53, as well as the previously mentioned TRBV4-1, in patient P2 was driven by the most expanded clones (fig. S11, D and E). Last, CD45^+^CD3^+^ cell proliferation upon stimulation with anti-CD3 or anti-CD3/CD28 antibodies and measured by the Click-iT EdU assay documented less responsive T cells in patient 4 (Fig. 7E). At the transcriptomic level, scRNA-seq performed on whole blood cells of patient 2 revealed an up-regulation of genes involved in calcium signaling, mitochondria and ER homeostasis, and cytoskeleton organization. There was also a down-regulation of genes involved in T cell activation. These deregulated pathways were specifically identified in the T cell subpopulations (Fig. 7, H and J).

## DISCUSSION

Given the ubiquity of calcium function in nearly every human cell, it was perhaps expected that a recent data-mining study reported that close to 2000 genes and 1500 human genetic disorders were linked to calcium signaling (*24*). As expected, many of these genes and related diseases are connected to the function of calcium channels and transporters in excitable cells, essentially neurons and (cardio)myocytes. In comparison, relatively few of these channelopathies have been documented to specifically affect the immune system (*25*), and these channelopathies are principally due to recessive or biallelic variants in *ORAI1*, *STIM1*, or the recently described regulatory protein *CRACR2A* (*26*).

Here, we presented a series of four unrelated patients carrying the same de novo missense variant in *ITPR3*. The variant did not overtly affect the gene’s transcription, yet it diminishes the expression of the protein. Experiments in patient-derived cells and gene-edited Jurkat cell lines further confirmed that this variant alone is responsible for and capable of disturbing intracellular calcium homeostasis and hence ultimately the clinical phenotype. Structural modeling clearly showed that the variant, R2524C, disrupts critical interactions with neighboring residues. In vivo structure function by mutagenesis further unveiled that residue 2524 is critical for the permeation to calcium, leading, perhaps as expected, to perturbation of mitochondrial homeostasis, a fact corroborated by proteomic, bulk, and scRNA-seq analyses and by the colocalization analysis of IP3R3 at ER-mitochondria contact sites, linking the identified IP3R3 variant to mitochondrial dysfunction. In addition, our multiomics analyses also indicate an impact of the IP3R3 variant on vesicle-mediated transport, which was recently suggested by Held *et al.* (*27*) who showed that IP3R3 depletion increases ER-to-Golgi vesicle trafficking. Vesicle-mediated Ca^2+^ transport might therefore represent a compensatory mechanism to the loss of Ca^2+^ flux via the IP3R pump. The clinical phenotype of the four patients allows recognizing a complex multisystemic disease including a severe immunodeficiency arising in most cases in the first year of life combining severe recurrent upper respiratory infection, otitis media, mastoiditis, and a host of other viral or bacterial infections. The immune phenotype seems restricted to the lymphoid lineage with mainly a CD4 lymphopenia originating from a diminution in naïve cell numbers and an increase in the memory compartment. This decreased ratio of naïve-to-memory cells might be explained by the association of a lower thymic output of naïve T cells with repeated antigenic stimulation during infection episodes that favor EM cell production. Neurological symptoms that could be categorized as Charcot-Marie-Tooth disease were identifiable in one patient. In addition, a host of other disorders are also carried by these patients certifying the pleiotropic nature of this variant. These include ectodermal dysplasia, neurodevelopmental delays, short stature, and hematological manifestations and are in line with low tissue specificity of IP3R3 mRNA and protein expression (https://proteinatlas.org). These non-immunological manifestations are in line with previous reports associating defects in calcium signaling related to the IP3R complex with neurological diseases (discussed elsewhere in Discussion).

As mentioned above, the identification of LoF and null variants in *STIM1* and *ORAI1* defined a nosological entity, the CRAC channelopathies. Whether *ITPR3*-based immunodeficiency disease belongs within CRAC channelopathies is at this point more a matter of semantics than medicine (although we believe that the observed phenotype is larger than those identified in CRAC channelopathies), yet it is of interest to briefly compare the phenotype of patients reported here with those suffering from *ORAI1* and *STIM1* null or LoF variants. The latter patients suffer from severe immunodeficiency disease, ectodermal dysplasia, autoimmunity, nonprogressive global muscle hypotonia, and mydriasis. A careful phenotypic comparison between our patients and patients with these channelopathies revealed many overlapping symptoms (perhaps as expected, as all three genes are key players in SOCE), yet there was some divergence (Table 3). Some features are readily comparable indeed. These included the fact that key symptoms appear within the first year of life and, in some instances, rapidly deteriorate (only to be rescued by HCT). Symptoms revealing a SCID-like disease are quite indistinguishable with notably recurrent infections. Of notable differences were the absence of generalized muscular hypotonia in our patients, as well as mydriasis. Furthermore, we observed no clinical or biological signs of autoimmunity in our patients. Last, in contrast to STIM1 or ORAI1 or a few other T cell deficiencies with a normal number of circulating lymphocytes (*8*), our patients were generally lymphopenic (data S1 and Table 1), and abnormally low T cell receptor excision circles (TREC) levels were detected in the only patient (patient 4) screened at birth for severe T cell lymphopenia. This marks an additional difference with ORAI1 and STIM1 deficiencies, as patients with these disorders have normal TREC levels at birth (*28*). Overall, these data indicate that IP3R3 R2524C affects thymopoiesis, which is not the case for ORAI1 and STIM1 deficiencies. The ectodermal phenotype of IP3R3 R2524C patients partially overlaps the phenotype of anhidrotic ectodermal dysplasia with immunodeficiency and *incontinentia pigmenti* due to *IKBKG*/*NEMO* variants, where patients display hypotrichosis, skin defects (hypo/anhidrosis and periocular hyperpigmentation), nail dysplasia, and tooth abnormalities, such as hypodontia and conical teeth (*29*). IP3R3 R2524C patients only show a mild phenotype of ectodermal dysplasia with no apparent hypodontia, facial skin hyperpigmentation, or nail dysplasia. In addition to ectodermal dysplasia, CRAC channelopathies (STIM1 and ORAI1 deficiencies) are also characterized by enamel defects, such as hypomineralized amelogenesis imperfecta (*7*), which was not observed in our series of IP3R3 R2524C patients. However, tooth shape abnormalities and hypopigmentation of the scalp hair or eyebrows were reported in three of our cases, and whitening of the nail beds was reported in one. Last but not least, unlike ORA1 and STIM1, it appears that ITPR3 defects could be detected through newborn screening programs assessing low TREC levels at birth. And again, as mentioned elsewhere in the manuscript, the phenotype in our patient seems broader than “classical” CRAC channelopathies as well as other ITPR deficiencies, hence defining a distinctive nosological entity.

**Table 3.** Comparison between patients suffering from CRAC channelopathies and those reported in this study (last column). AR, autosomal recessive; AD, autosomal dominant; CID, combined immunodeficiency; Igs, immunoglobulins; LRI, lower respiratory tract infections; N/A, not available; PHA, phytohemagglutinin; PMA + iono, phorbol myristate acetate + ionomycin; RTE, recent thymic emigrants; SCID, severe combined immunodeficiency.

	*ORAI (* *81* *)*	*STIM1 (* *81* *)*	*CRACR2A (* *26* *)*	*ITPR3*
**Clinical phenotype**				
Heredity	AR	AR	AR	AD
Immunological phenotype	SCID, CID	SCID, CID	Later onset, chronic diarrhea, recurrent LRI	SCID, CID
Ectodermal dysplasia	+	+	−	+
Myopathy	+	+	−	−
Autoimmunity	+	+	−	−
Others	Facial dysmorphism, osteopetrosis, hepatosplenomegaly, bleeding tendency, bronchiectasis, mydriasis	Facial dysmorphism, osteopetrosis, hepatosplenomegaly, bleeding tendency, lymphoproliferation, mydriasis	Bronchiectasis	Charcot-Marie-Tooth disease, lymphoproliferation
**T cells**				
T cell numbers	Normal	Normal		CD4 lymphocytopenia
	↓ Naïve CD4^+^	↓ Naïve CD4^+^	↓ Naïve CD4 T	↑ Memory
	↑ Memory CD4^+^	↑ Memory CD4^+^	↓ T_reg_	↓ Naïve CD4^+^
	↓ T_reg_	Abnormal T_regs_		↓ Naïve CD8^+^
	↓ iNKT	↓ iNKT		↓ RTE
T cell response to stimuli	Anti-CD3 ↓	Anti-CD3 ↓	Anti-CD3 ↓	Anti-CD3 ↓
	PHA ↓	PHA ↓	PHA ↓	PHA ↓
	PMA + iono	PMA + Iono ↓	PMA + Iono ↓	
	Normal to ↓			
TCR repertoire	N/A	Normal	Normal	↓ Diversity
**B cells**				
B cell numbers	Normal	Normal	Normal	Normal to ↓
	↓ Unswitched memory	↓ Unswitched memory	↓ Switched memory	↓ Switched memory
				↓ Unswitched memory
Igs	Normal to ↑	Normal to ↑	Pan-hypogammaglobulinemia	Normal
	Impaired specific Igs	Impaired specific Igs		
**Cytokine production**	Impaired	Impaired	Impaired	Impaired

TCR repertoire skewing has been observed in patients with certain inborn errors of immunity (*8*, *30*, *31*). Decreased diversity of the T cell repertoire (fig. S11A) and prominent clonotypic expansions (fig. S11B) were observed in both patients compared with age-matched controls. Despite alteration in clonal size distribution, TRA and TRB rearrangement analysis did not show qualitative repertoire skewing (fig. S11). Future studies may help define the relative contribution of impaired thymic output and homeostatic T cell proliferation versus infection-driven clonotypic expansions in driving skewing of TCR repertoire in IP3R3 R2524C patients.

The finding of the same variant in four independent patients from different ethnic backgrounds is intriguing. The fact that *ITPR3* is encoded on chromosome 6 almost within the boundaries of the major histocompatibility complex (MHC; HLA in man, the most polymorphic region of the human genome), approximately 300 kb centromeric to the “official” border of the HLA locus (defined by the *TAPBP* gene), might suggest that this variant has been fixed on a particular ancestral MHC haplotype, because of the well-known strong linkage disequilibrium within the HLA complex (*32*, *33*). The HLA typing results of all four patients provides partial support for this hypothesis, as there are several identical alleles at some HLA loci (*HLA-A*, *HLA-C*, and *HLA-DRB4*) between the first two patients (fig. S13). The further identification of patients and variants could settle this issue.

IP3R being a key component of intracellular calcium homeostasis, it is therefore of interest to compare our findings at present to genotype-phenotype correlations for other variant(s) identified in *ITPR1*, *ITPR2*, and *ITPR3* genes in man, or genetic engineering of the same variants in mouse, and such in order perhaps to understand whether there are any differential functionality for each of these three genes, their variants, and their products. Although the literature is replete with reports of different variants in the same gene leading to very different phenotypes, it is of note that, thus far, the vast majority of *ITPR1*, *ITPR2*, and *ITPR3* variants reported in man have been almost exclusively linked to neurological disorders (although the situation is different in mouse; see below). These include Gillespie syndrome (*34*), spinocerebellar ataxia type 15 (*35*) and type 29 (*36*) for *ITPR1*; generalized, isolated anhidrosis for *ITPR2* (hence without other ectodermal manifestations, e.g., hair and teeth) (*37*); and autosomal dominant demyelinating Charcot-Marie-Tooth disease type 1J for *ITPR3* (*38*). In mice, each IP3R component has been genetically deleted whether individually or in combination with others. The obtained phenotypes are quite diverse. Whereas there are obvious overlaps between human and mouse phenotypes for *ITPR1* and *ITPR2*, there are none for *ITPR3*. Here is a summary: *ITPR1* KO die in utero or soon after birth while displaying ataxia and severe seizures (*39*), a neurological phenotype not dissimilar with those seen in man (see above and OMIM #147265); *ITPR2* KO live an extended life span (*40*), along with reported cardiac rhythmogenesis abnormalities (*41*) but perhaps more remarkably with sweat gland abnormalities matching perfectly anhidrosis found in man (see above and OMIM #600144); whereas *ITPR3* KO mice were reported to have abnormal taste perception (*42*) or yet alopecia (*43*), none matching findings in man (see above and OMIM #147267). Double KOs add some complexity to the observed phenotypes, i.e., not unexpectedly *ITPR1* and *ITPR2* double KO mice also died in utero along with abnormal myocardium (*44*); *ITPR1* and *ITPR3* double KO mice while also lethal in utero showed abnormal vasculature at the materno-embryonic interface (*45*); whereas *ITPR2* and *ITPR3* double KO show defects in exocrine function (*46*) and olfactory mucus secretion (*47*). KO of all three genes being certainly lethal, conditional genetic ablation of all three *ITPRs* in selective immunocytes led to abnormal development of thymocytes (*48*) and B lymphocytes (*49*), yet no broad immunodeficiency has been identified in these multiple animal models of IP3R deficiency. With regard to the very variant described in this manuscript, a recent paper reported two patients suffering from dissimilar immune phenotypes, reportedly due to compound heterozygous variants in *ITPR3* (*12*). There are some notable differences between this publication and our work that we believe merit attention. Neumann and colleagues (*12*) patient 1 harbors the same variant as our four patients, i.e., R2524C, and such in a de novo manner (similar to our patients). However, this same patient inherited a second variant from his father, c.5549G>A, p.Arg1850Gln (R1850Q), which was also found in their patient 2 and his father. Patient 2 also inherited a second private variant from his mother, a c.4882T>C, p.Phe1628Leu (F1628L) (table S1). These colleagues therefore concluded that the causality of this disease is because of compound heterozygous (biallelic) variants in *ITPR3*. However, c.5549G>A, p.Arg1850Gln (R1850Q) (also present in our patient 1 and his father; see table S1) is a common genetic variant with a minor allele frequency (MAF) of more than 6% in public databases. In addition, there are 763 homozygous individuals in gnomAD that are likely not affected. The hypothesis of R1850Q being a hypomorphic allele is not likely either, as we identified 25 individuals in CENTOGENE’s database that were heterozygous for this variant and at least one additional rare variant in the same gene and none show a phenotype matching the patients reported here. We believe therefore that on the basis of data presented here, the autosomal recessive (compound heterozygosity) interpretation needs reappraisal for the following reasons: (i) The R1850Q variant proposed to contribute to the phenotype in both patients is too frequent, as MAF is 0.06 in the pooled gnomAD v2 population. Its allele frequency is even higher (i.e., close to 10%) in the European population and population of European descent to which both patients belong (0.098 1K EUR; 0.099 ESP EA; 0.096 gnomAD non-finish European), (ii) the variant is predicted to be tolerated/benign by most of the predictive tools (scale-invariant feature transform, PolyPhen, MutationTaster, GERP, and ClinVar) (see table S1), and (iii) the phenotype of patient 2 in Neumann *et al.* (*12*) is different from that of any of our patients (and their patient 1). Of equal interest is also the publication by Rönkkö and co-workers (*9*), which documents the potential pathogenicity of the same variant reported here (R2524C) in a case of Charcot-Marie-Tooth disease with a de novo inheritance mode. It is of note that the authors do not report any immunological phenotype in their patient, further proof of the pleiotropic nature of the variant.

Furthermore, at least three lines of evidence are in favor of a dominant-negative mode of action for R2524C. First, despite the fact that in both patient and KI cells both (healthy and mutated) *ITPR3* alleles are equally transcribed (as documented by RNA-seq), the total amount of IP3R3 protein is diminished as seen in Western blotting and immunofluorescence. Second, the presence of 59 LoF variants in gnomAD, associated with a pLI score of 0 and *Z* score of 4.55 (*50*), and that of over 200 individuals with heterozygous putative LoF variants (over 50 are reported to be healthy) in CENTOGENE’s Biodatabank are strongly against a haploinsufficiency mechanism. Third, Jurkat cells transfected with the R2524C mutated *ITPR3* gene show an impaired calcium flux as compared to cells transfected with the WT gene (Fig. 3, E and F).

Last, although the number of patients identified with this disease is presently limited, given the unicity (thus far) of the pathogenic variant, one can nonetheless already conclude as to a heterogeneous clinical symptomatology for this syndromic immunodeficiency, including the entrance to the disease. These include, besides the classical severe iterative infections, newborn screening for SCID or yet hematopathological manifestations which could be classified as a HLH-like syndrome. Further along the course of the disease, one patient developed Charcot-Marie-Tooth disease, three showed short stature, and lastly three displayed ectodermal dysplasia.

In conclusion, while calcium signaling is critical for the function of nearly every immunocyte, only three genetic defects have been thus far identified to lead to combined immunodeficiency. Here, we report a channelopathy leading to a complex immune deficiency along with multisystemic manifestations. The identification of ITPR3-related immune dysregulation further diversifies the spectrum of calcium-related immune disorders and has, besides further understanding of calcium homeostasis and the function of the immune system, obvious etiological, diagnostic, nosological, and therapeutic relevance.

## MATERIALS AND METHODS

### Subjects and study approval

The subjects reported in this study were members of four unrelated families of French, Israeli, and North American origins. In all four families, the parents and other siblings were healthy. All sequencing (whole-exome and Sanger) was performed after written informed consent for either clinical sequencing and/or institutional review board–approved research sequencing in medical genetics of Nantes University Hospital (Ministère de l'enseignement supérieur et de la recherche DC-2017-2987, Comité de protection des personnes Ouest IV 06/15). Consent for published images was obtained from parents. All procedures were performed in accordance with the Helsinki Declaration.

### WES and Sanger sequencing

Genomic DNA was isolated from patient and relatives’ peripheral blood or buccal swabs using the standard protocols. WES of family 1 (I.1, I.2, and II.1) was performed on a NextSeq 500 sequencer (Illumina, San Diego, CA) as previously described (*51*). WES of family 2 (I.1, I.2, II.2, and II.3) was performed at CENTOGENE (Rostock, Germany) as previously described (*52*). WES of family 3 (I.1, I.2, and II.1) was performed at GeneDx (Gaithersburg, MD) using the IDT xGen Exome v1 enrichment capture kit (Integrated DNA Technologies Inc., Coralville, IA). WES of family 4 (I.1, I.2, and II.1) was performed at Blueprint Genetics (Espoo, Finland) using a hybridization-based target capture method, followed by NovaSeq sequencing (Illumina, San Diego, CA). All WES were reanalyzed according to the following filtering criteria where we focused on protein-altering variants (missense, nonsense, and indels) covered by at least 10 reads, with alternative allele frequencies below 0.001 in the 1000 Genomes Project, the Genome Aggregation Database (Exomes gnomADv2.1.1), and our internal exome database including ~1000 exomes of inborn errors of immunity. To identify potential causal variants, we further filtered for de novo variants, i.e., heterozygous in the patients and absent (WT) in both parents (alternative read count above 3 for patient and below 3 for parents with a percentage of alternative depth above 25% and below 75% for patients). In families 1, 2, and 3, a single variant met these filtering criteria: *ITPR3*; c.7570C>T, p.Arg2524Cys. Two variants remained after filtering in family 4, i.e., *ITPR3*; c.7570C>T, p.Arg2524Cys, hence common to all four families (Table 2), and a missense variant in *MSTO1*; c.481A>G, p.Ile161Val (table S1). Ensembl Variant Effect Predictor, encompassing various bioinformatic tools, was used to annotate the variant and attribute predictive scores of pathogenicity (Table 2 and table S2). To exclude the presence of higher frequency, de novo, homozygous or compound heterozygous variants in *ITPR3*, its homologs *ITPR1* and *ITPR2*, as well as *ORAI1* and *STIM1*, which are known to cause CRAC channelopathies, we analyzed all protein-altering variants regardless of allele frequency and applied solely filters on sequencing depth (minimum of 10 reads) and inheritance (variants homozygous in parents and healthy siblings or heterozygous in patient and both parents have been excluded). All variants passing this conservative filtering approach, including the candidate variant ITPR3 p.Arg2524Cys, are described in table S1. Raw exome data (FASTQ files) are available at the National Center for Biotechnology Information’s Sequence Read Archive under accession no. PRJNA913788.

For families 1 and 2, the *ITPR3* candidate variant (c.7570C>T) was also confirmed by capillary Sanger sequencing using 5′-CAACCTGAGTCCTATCTTGCCCCAG-3′ and 5′-GGAAGGGCCAAACCGCTACTG-3′ primers, in patients and relatives using the Big Dye Terminator Kit v3.1 and an ABI PRISM 3730xl sequencer (Thermo Fisher Scientific, Waltham, MA). Data were analyzed using the SeqScape software (Thermo Fisher Scientific, Waltham, MA).

### HLA typing

Upon exome analysis of the *ITPR3* family members, the sequencing data were available in BAM (Binary Alignment Map) format, one file per sample. From each BAM file, we extracted all the reads aligned to the MHC region along with the unmapped reads using the SAMtools 1.14 software suite (*53*). The extracted reads were next stored in FASTQ format, two paired-end FASTQ files per sample, for typing analysis. The typing of each sample was first inferred using the HLA-HD software (*54*) and confirmed with the HISAT-genotype tool (*55*). As a result, high-resolution typing information became available for each member of the families for the genes *HLA-A*, *HLA-B*, *HLA-C*, *HLA-DRB1*, *HLA-DQB1*, and *HLA-DPB1*.

### Structural modeling

For modeling purposes, we used the tetramer complex structure of IP3R3 in the Protein Data Bank (PDB) (*56*) as a template, PDB 6drc (*5*). The Swiss-model (*57*) webserver was used to build the WT IP3R3 protein complex structure. Mutagenesis analysis was performed using “mutagenesis” add-on in the PyMOL (version 2.0 Schrödinger LLC n.d. “The PyMOL Molecular Graphics System”; https://pymol.org) program. The backbone-independent rotamer library was used for modeling the mutated side chains.

### ITPR3 baculovirus generation

All constructs used were N-terminally tagged with 10xHis followed by mVenus (WT-IP3R3) or enhanced green fluorescent protein (EGFP; R2524X variant constructs) followed by human rhinovirus 3C protease cut site followed by the ITPR3 coding sequence. Baculoviruses were generated as previously described (*5*). Briefly, DH10Bac cells were transformed with plasmid constructs to generate and purify bacmids using isopropanol precipitation. One hundred to 200 μg of purified bacmid was then resuspended in 1 ml of dH_2_O, incubated with 400 μg of 25,000 MW polyethyleneimine (PEI; Polysciences, Warrington, PA), and heated at 55°C for 30 min followed by a 15-min incubation at room temperature. The DNA:PEI complex was then added to 50 ml of Sf9 cells (Expression Systems, Davis, CA) at 1 × 10^6^ cells/ml grown in suspension at 27°C. The Sf9 Expression Systems’ ESF 921 media was supplemented with 1% penicillin/streptomycin and 5% fetal bovine serum (FBS) to stabilize the virus generated. Virus titer was amplified using a 1:10 virus:cell culture ratio up to P3 generation and separated from cell debris by centrifugation at 4000 rpm for 10 min. Titer of the P3 virus was not quantified, and thus expression levels of different baculoviruses cannot be compared.

### IP_3_R-null cell culture

HEK293T-IP_3_R-null cells were obtained through Kerafast (Kerafast, Boston, MA) (*58*) and cultured as previously described (*5*). Briefly, cells were grown to a confluency of ∼75 to 80% on 100 mm by 20 mm tissue culture–treated dishes in Dulbecco’s modified Eagle’s medium (DMEM) supplemented with 10% FBS, penicillin (100 U/ml), and streptomycin (100 mg/ml) at 37°C with 5% CO_2_. For imaging, cells were then split in a 1:4 ratio and plated on poly-d-lysine–coated, 35-mm-diameter, optical quality glass-bottom culture dishes (World Precision Instruments, Sarasota, FL) and incubated for ∼18 to 24 hours. At ∼60% confluency, cells were transduced with 200 μl of P3 baculovirus followed by incubation at 37°C and 5% CO_2_ for another 24 hours.

### Fluorescence detection size exclusion chromatography of human IP3R3 constructs

HEK293T-IP_3_R-null cells were grown on six-well tissue culture–treated flat-bottom plates in DMEM supplemented with 10% FBS, penicillin (100 U/ml), and streptomycin (100 mg/ml) at 37°C with 5% CO_2_. At ∼60% confluency, cells were transduced with 200 μl of P3 baculovirus or with a mock transduction followed by incubation at 37°C and 5% CO_2_ for another 24 hours. After transduction, 2 ml of cells was pelleted, and membrane proteins were solubilized as previously described (*5*). Briefly, cell pellets were solubilized in 200 μl of 0.488 mM digitonin (Millipore Sigma, Burlington, MA), 150 mM NaCl, 20 mM Hepes (pH 7.5), 1 mM phenylmethylsulfonyl fluoride, aprotinin (2.5 μg/ml; Sigma-Aldrich, St. Louis, MO), leupeptin (2.5 μg/ml; Alfa Aesar, Heysham, Lancashire, UK), pepstatin A (10 μg/ml; GoldBio, St. Louis, MO), 0.5 mM 4-benzenesulfonyl fluoride hydrochloride (EMD Millipore, Burlington, MA), and a few flakes of lyophilized deoxyribonuclease (Worthington Biochemical, Lakewood, NJ). The resulting cell lysate was centrifuged at 21,000*g* for 40 min. Fluorescence size exclusion chromatography was performed on 80 μl of the supernatant with a Superose 6 Increase column and gel filtration buffer containing 150 mM NaCl, 50 mM tris-HCl (pH 8.0), 0.488 mM digitonin, and 2 mM dithiothreitol (DTT) to assess retention volumes of the fluorescently tagged proteins.

### Ca^2+^ imaging and data processing

Ca2^+^ imaging and data analysis of IP3R3 and variant constructs were performed as previously described (*5*). Twenty-four hours after baculovirus transduction, cells were gently washed with live cell imaging solution (catalog no. A14291DJ, Invitrogen, Carlsbad, CA) followed by incubation for 1 hour at 37°C and 5% CO_2_ in 1800 μl of imaging buffer containing 3 μM Cal-520-AM (#21130AAT, Bioquest, Pleasanton, CA). Cal-520-AM–loaded cells were removed from the CO_2_ incubator and equilibrated at room temperature for 5 min before IP_3_ stimulation by the addition of 200 μl of 1 mM carbachol (#L06674-06, Alfa Aesar, Haverhill, MA), a Gαq-coupled M3 muscarinic receptor agonist. Carbachol was added at least 10 mm away from the imaging site and allowed to diffuse to a final concentration of 100 μM. Movies of carbachol-induced Ca^2+^ release in cells were collected at 20× with LD Plan-Neofluar 20X/0.4 Korr M27 objective, for 10 min, at 3 × 3 binning (912 × 736 pixels after binning), with an exposure time of 250 ms on a Zeiss Axio Observer D1 inverted phase-contrast fluorescence microscope equipped with an Axiocam 506 Mono camera (Zeiss, Oberkochen, Germany). Cal-520-AM imaging was carried out by exciting the sample at 493 nm and monitoring emission at 515 nm using an X-Cite Series 120Q illumination system and Zeiss filter set 38 HE. Ca^2+^ imaging movies were processed using ImageJ (*59*), Fiji (*60*), and MathWorks MATLAB 9.12.0.1884302 (R2022a) to extract Cal-520-AM fluorescence traces from individual cells. Movie stacks were background-subtracted with a 200-pixel rolling ball radius in ImageJ. Maximum intensity projection of the stack was used to generate a difference of Gaussian image, which was used for edge detection and cell segmentation using MATLAB’s Image Processing Toolbox. Traces were then extracted from segmented cells, smoothed over 41 frames using a Savitzky-Golay filter of polynomial order 2, and baseline adjusted using the linear method of MATLAB’s 1-D data interpolation function with a custom MATLAB script called Baseline Fit (*61*). The data reported are from three independent biological replicates.

### HDF cell culture

HDFs were generated from a skin biopsy of patient 1 following routine procedures. Healthy control HDFs were purchased from Lonza (Morrisville, NC) and Promocell (Heidelberg, Germany). HDFs were maintained in DMEM GlutaMAX Gibco (Thermo Fisher Scientific, Waltham, MA) supplemented with 10% heat-inactivated FBS (PAN BIOTECH, Dutscher, Bernolsheim, France), penicillin (50 U/ml), and streptomycin (50 μg/ml; Thermo Fisher Scientific, Waltham, MA).

### Establishment of Jurkat *ITPR3* KI and KO as well as *ITPR2* KO cell lines

We performed CRISPR-Cas9 *ITPR3* KI and KO and *ITPR2* KO using a plasmid containing both Cas9 and single guide RNA (sgRNA) expression cassettes. The Cas9 gene is human codon–optimized and under the control of SV40 enhancer and chicken β*-actin* promoter. Cas9 N terminus was fused to 3xFLAG and SV40 NLS and C terminus to a nucleoplasmin NLS followed by a T2A self-cleaving peptide and EGFP. The target sequences for sgRNAs (sgRNA *ITPR3*: 5′-CTCACTACGCAGGTCAGCGA-3′; sgRNA *ITPR2*: 5′-GTTAGTGGATGACAGATGTG-3′) were designed in proximity of the variant in *ITPR3* (c.7570C>T) and in exon 2 of *ITPR2* using the CRISPOR (*62*) webtool (http://crispor.tefor.net/). Forward and reverse complement sgRNA target sequences extended by Bbs I restriction sites were annealed (95° to 1°C/min) and inserted by Bbs I digestion-ligation downstream of the U6 promoter and upstream adjacent to the guide RNA scaffold sequence. The Jurkat cell line was grown at 37°C under 5% CO_2_ and maintained in RPMI 1640 Gibco Medium (Thermo Fisher Scientific, Waltham, MA), penicillin (50 U/ml), and streptomycin (50 μg/ml; Thermo Fisher Scientific, Waltham, MA), which was supplemented with 10% FBS (PAN BIOTECH, Dutscher, Bernolsheim, France). Cells were transfected using the Neon Transfection System (Thermo Fisher Scientific, Waltham, MA). For a single 100-μl Neon Tip, 2 × 10^6^ cells, 5 μg of plasmid DNA, and 2 μM single-stranded donor oligos for homology-directed repair for *ITPR3* KI (5′-GAACCTCATCTTTGGGGTAATCATCGACACATTCGCTGACCTGTGTAGTGAGAAGCAGAAGAAGGAGGAGATTCTTAAGACGA-3′, Integrated DNA Technologies Inc., Coralville, IA) were used according to the manufacturer’s instructions. Transfected cells were seeded onto six-well plates in medium supplemented with 20 μM homology-directed repair rate enhancer (Integrated DNA Technologies Inc., Coralville, IA). After 48 hours, GFP-positive cells were sorted using a FACSAria Fusion (BD Biosciences, Franklin Lakes, NJ) onto 96-well plates. Upon 2 weeks of clonal amplification, DNA was extracted using the standard protocols, and CRISPR-Cas9 editing was analyzed by Sanger sequencing as described above. Two WTs (WT1 and WT2), one heterozygous *ITPR3* c.7570C>T KI, and two KOs (KO1 and KO2) were selected for *ITPR3* and one KO for *ITPR2*.

### Measurement of cytoplasmic Ca^2+^

HDFs were detached from dishes with phosphate-buffered saline (PBS) without Ca^2+^ and Mg^2+^ Gibco (Thermo Fisher Scientific, Waltham, MA) supplemented with 1 mM EDTA (Invitrogen, Carlsbad, CA), and CRISPR-Cas9–edited Jurkat cells were harvested by resuspension. For both cell types, cells were centrifuged at 250*g* for 10 min and resuspended at 1 × 10^6^ cells/ml in Hanks’ balanced salt solution (HBSS) with Ca^2+^ and Mg^2+^ Gibco (Thermo Fisher Scientific, Waltham, MA) containing 1 μM Indo-1-AM (Invitrogen, Carlsbad, CA) prediluted in an equal volume of Pluronic F-127 (Invitrogen, Carlsbad, CA), for 30 min at 37°C. After washing, cells were incubated for an additional 30 min at 37°C in HBSS with Ca^2+^ and Mg^2+^. Cells were washed and seeded in 96-black-well plates in HBSS with Ca^2+^ and Mg^2+^. Fluorescence was acquired on a Varioskan LUX (Thermo Fisher Scientific, Waltham, MA) at 340 nm for excitation and at 400 nm and 480 nm for emission of the Ca-bound and Ca-free Indo-1, respectively. Cellular calcium flux was stimulated by adding 1 μM ionomycin (Merck, Burlington, MA) or 1 μM thapsigargin (Invitrogen, Carlsbad, CA), in the presence of 5 mM EGTA (Alfa Aesar by Thermo Fisher Scientific, Waltham, MA). The signal was calculated as the ratio of Ca^2+^-bound to Ca^2+^-free fluorescence. Analysis in patient 4 was performed using freshly isolated PBMCs upon a Ficoll gradient from whole blood collected in sodium heparin tubes. Total PBMCs were stained for extracellular markers with anti-CD45 and anti-CD4 antibodies for 10 min in the dark at room temperature. Cells were washed in PBS and stained with the calcium indicator Fluo-4 AM (20 μM; catalog no. F14201, Thermo Fisher Scientific, Waltham, MA) and 4 μM probenecid (Invitrogen, catalog no. P36400) for 30 min at 37°C. Cells were then resuspended in PBS, and aliquots were prepared with 0.5 × 10^6^ cells per tube. To activate T cells through the TCR, cells were preincubated with anti-CD3 (10 μg/ml; catalog no. 16-0037-81, Thermo Fisher Scientific, Waltham, MA) and anti-CD28 (10 μg/ml; catalog no. 16-0289-81, Thermo Fisher Scientific, Waltham, MA) antibodies for 30 min on ice. Cells were washed with HBSS containing calcium and magnesium (catalog no. 14025-092, Gibco, Thermo Fisher Scientific, Waltham, MA) to replenish intracellular calcium stores and then washed again in calcium- and magnesium-free HBSS (catalog no. 14175-095, Gibco, Thermo Fisher Scientific, Waltham, MA). Cells were resuspended in calcium- and magnesium-free HBSS before assessment by flow cytometry with an LSRFortessa cell analyzer (BD Biosciences, Franklin Lakes, NJ). The kinetics of intracellular Ca^2+^ were recorded by collecting baseline levels for 180 s, followed by calcium reconstitution using 2 M CaCl_2_ (catalog no. BP510-500, Thermo Fisher Scientific, Hampton, NH). For TCR activation in preincubated CD3/CD28 cells, goat anti-mouse IgG linker antibody (10 μg/ml; catalog no. ab6708, Abcam, Cambridge, UK) was added after 60 s of baseline acquisition. Data are presented as the change of geometric mean of Fluo-4 fluorescence of CD45^+^CD4^+^ T cell population of each sample over time (seconds).

### Transient transfection of Jurkat cells and calcium measurement

Jurkat cells were transfected with *ITPR3*-expressing plasmids using Lipofectamine 3000 (Invitrogen, Carlsbad, CA) following the manufacturer’s instructions. Briefly, 2 × 10^6^ cells per well were seeded in a six-well plate 1 day before the transfection. Ten micrograms of plasmids was mixed with 7.5 μl of Lipofectamine 3000 (Invitrogen, Carlsbad, CA) in Opti-MEM medium (Invitrogen, Carlsbad, CA) and incubated for 15 min before drop wisely added to the seeded cells. Forty-eight hours after the transfection, cells were collected, counted, and washed with PBS. Cells were then resuspended in PBS + 1% FBS to a concentration of 2 to 5 × 10^6^ cells/ml. Indo-1-AM (Invitrogen, Carlsbad, CA) prediluted in an equal volume of Pluronic F-127 (Invitrogen, Carlsbad, CA) was added to achieve a concentration of 1 μM and incubated in 37°C for 45 min. During the incubation, the cells were vortexed to remix every 15 min. After washing, cells were resuspended in 1.5 ml of HBSS without Ca^2+^ and Mg^2+^ (Gibco, Thermo Fisher Scientific, Waltham, MA) and stocked on ice. Five minutes before measurement, 500 μl of cells was transferred to a new fluorescence-activated cell sorting (FACS) tube and incubated in a 37°C water bath to rewarm the cells. The warmed cells were then analyzed using a Cytek Aurora 5L spectral flow cytometer (Cytek Biosciences, Fremont, CA). The Indo-bound, indo-unbound, and the mVenus signals were recorded in the UV-2, UV-6, and B-1 channels, respectively. After 1 min of baseline recording, cellular calcium flux was stimulated by adding ionomycin (Merck, Burlington, MA) or thapsigargin (Invitrogen, Carlsbad, CA) to a final concentration of 1 μM. Results were analyzed using FlowJo v10 (BD Biosciences, Franklin Lakes, NJ).

### Relative quantification of *ITPR3* transcripts

Total RNA was extracted from PBMCs, HDFs, and total blood sampled in PAXgene Blood RNA tubes (BD Biosciences, Franklin Lakes, NJ) using, respectively, the RNeasy Mini Kit and the QIAamp RNA Blood Mini Kit (Qiagen, Hilden, Germany) and reverse-transcribed using Maxima H Minus cDNA Synthesis Master Mix (Thermo Fisher Scientific, Waltham, MA) according to manufacturers’ instructions. Real-time quantitative PCR was performed in a total volume of 20 μl using the PowerTrack SYBR Green Master mix kit (Thermo Fisher Scientific, Waltham, MA) and the following gene-specific primers: *ITPR3*, 5′-AACTACCTGGCTGCTGAGGA-3′ and 5′-CGAAAGAGTCGGTTTTCTGC-3′; *GAPDH*, 5′-GGTGAAGGTCGGAGTCAACGGA-3′ and 5′-GAGGGATCTCGCTCCTGGAAGA-3′; β*-actin*, 5′-CCAACCGCGAGATGACC-3′ and 5′-GATCTTCATGAGGTAGTCAGT-3′. After an initial denaturing at 96°C for 10 min, the temperatures and incubation times used for cycling were 95°C for 10 s and 60°C for 30 s using a QuantStudio 3 real-time PCR machine (Thermo Fisher Scientific, Waltham, MA). Melting curve analysis was performed to assess the specificity of the PCR products, and relative expression results were obtained using the 2^–∆∆Ct^ method with *GAPDH* and β*-actin* as internal reference genes.

### Bulk RNA-seq

For whole transcriptome analyses, RNA was isolated from PBMCs, HDFs, and total blood sampled in PAXgene Blood RNA tubes (BD Biosciences, Franklin Lakes, NJ) using, respectively, the RNeasy Mini Kit and the QIAamp RNA Blood Mini Kit (Qiagen, Hilden, Germany). RNA integrity was evaluated on an Agilent Bioanalyzer 2100 using an Agilent RNA 6000 Pico Kit (Agilent Technologies, Santa Clara, CA). Globin transcripts were depleted from RNA extracted from total blood using a GLOBINclear-Human kit (Thermo Fisher Scientific, Waltham, MA). Total RNA-seq libraries were prepared with the SMARTer Stranded Total RNA-Seq Kit v2 - Pico Input Mammalian (TaKaRa, Kusatsu, Japan) according to the manufacturer’s protocol. Libraries were paired-end sequenced (2 × 75 bp) on a NextSeq 500 and (2 × 100 bp) on a NextSeq 2000 (Illumina, San Diego, CA) and analyzed as previously described (*51*). As indeed described previously (*51*), for each sample, quality control was performed and assessed using the next-generation sequencing (NGS) Core Tools FastQC. Sequence reads were mapped using STAR (*63*). Unmapped reads were remapped with Bowtie2 (*64*) using a very sensitive local option to optimize the alignment. The total mapped reads were finally available in Binary Alignment Map format for raw read count extraction. Read counts were determined by the HTseq-count tool of the Python package HTSeq (*65*) with default parameters to generate an abundance matrix. Last, differential analyses were performed using the edgeR (*66*, *67*) package of the Bioconductor framework for RNA-seq data. Up- and down-regulated genes between the patients and controls were selected on the basis of the adjusted *P* value (<0.05) and the fold change (>1.5). Raw RNA-seq data have been deposited in the EMBL-EBI ArrayExpress archive (accession no. E-MTAB-12432).

### TCR RNA-seq

For TCR α and β chain analyses, RNA was isolated from PBMCs using the RNeasy Mini Kit (Qiagen, Hilden, Germany) and from total blood sampled in PAXgene Blood RNA tubes (BD Biosciences, Franklin Lakes, NJ) using the QIAamp RNA Blood Mini Kit (Qiagen, Hilden, Germany). RNA integrity was evaluated on an Agilent Bioanalyzer 2100 using an Agilent RNA 6000 Pico Kit (Agilent Technologies, Santa Clara, CA). Libraries were prepared using the SMARTer Human TCR a/b Profiling kit (TaKaRa, Kusatsu, Japan) according to the manufacturer’s protocol, and paired-end sequencing (2 × 300 bp) was performed using a MiSeq instrument (Illumina, San Diego, CA). FASTQ files were processed by Cogent NGS Immune Profiler Software v1.0. In short, the reads were split by matching read sequence to different receptor chains. Short reads (<30 bp) and ambiguous reads that matched multiple receptor chains were excluded. The reads were then grouped into molecular identifier groups using unique molecular identifiers (UMI) and collapsed to generate other FASTQ files. The resulting sequences were aligned to VJ sequences to identify clonotypes and to report statistics such as numbers, percentage, nucleotide, and amino acid sequences of the different clonotypes. The statistics were further analyzed using the immunarch R-Package (immunomind/immunarch: Immunarch 0.9.0 | Zenodo) (*65*). The heatmaps represent V-J segments usage. For each sample, the V-J genes were ordered from 5′ to 3′ and counted taking into account clonal expansion. The counts were then normalized by the total count per sample. Raw data (FASTQ files) are available at the National Center for Biotechnology Information’s Sequence Read Archive under accession no. PRJNA913788.

### Single-cell RNA-seq

Following treatment of EDTA whole blood with 1× red blood cell lysis solution (Miltenyi Biotec, Bergisch Gladbach, Germany), cells were washed and centrifuged at 500*g* before being resuspended in PBS containing 2% FBS. Cell counting was performed with Trypan Blue and a Countess II Automated Cell Counter (Thermo Fisher Scientific, Waltham, MA). Following quality control (cell viability, >70%), the single-cell suspension was loaded at a volume to target of approximately 5000 cells per sample onto a Chromium Controller (10X Genomics, Pleasanton, CA) for Gel bead-In-EMulsions (GEM) generation according to manufacturer’s instructions (10X Genomics, Pleasanton, CA). After GEM generation, single index libraries were generated using the Chromium Single Cell 3′ Library and Gel Bead kit v3.1, according to manufacturer’s instructions (PN 1000121, 10X Genomics, Pleasanton, CA). Size distribution and concentration of cDNA and final libraries were verified on an Agilent Bioanalyzer High Sensitivity chip (Agilent Technologies, Santa Clara, CA), and the concentrations of the libraries were measured using a Qubit Fluorometer (Thermo Fisher Scientific, Waltham, MA) and the Qubit dsDNA HS Assay Kit (Thermo Fisher Scientific, Waltham, MA). Libraries were paired-end sequenced on a NovaSeq 6000 instrument (Illumina, San Diego, CA) according to the manufacturer’s guidelines with a sequencing depth of at least 30,000 reads per cell. Raw sequencing data were processed using Cell Ranger analysis pipeline (version 6.1.2) (10X Genomics, Pleasanton, CA). The “mkfastq” command was used to generate FASTQ files, and the “count” command was used to generate raw gene-barcode matrices aligned to the 10X Genomics GRCh38 Ensembl build 84 genome (version 1.2.0). The h5 file of each sample was then processed with Partek Flow analysis software (Partek Incorporated, Chesterfield, MO) for further analysis.

Filtering was conducted by retaining cells that had UMI between 500 and 13,800, expressed genes between 200 and 2800 genes, and had a mitochondrial content less than 10%. This resulted in a total of 10,973 cells (4152 cells for patient 2 and 6821 cells for the control). After this step, we filtered the set of genes, on one hand by a noise reducing filter (removing all features whose value was less than or equal to zero in 99% of the cells) and all the features expressed on the X and Y chromosomes. Sample sequencing depth was normalized to count per million and log-transformed after adding a pseudocount of 1.

Principal components analysis was performed on the filtered feature-by-barcode matrix. We used Harmony for data integration to account for patient-specific effects (*68*). Graph-based clustering was performed for each superset using the Louvain algorithm with a resolution of 0.5. Uniform manifold approximation and projection (UMAP) was based on the batch-corrected Harmony components.

Cell populations were identified using Azimuth (Azimuth; https://hubmapconsortium.org/), a tool for reference-based single-cell analysis (*69*). On the basis of the flow cytometry results, the fact that the time between venipuncture and analysis was 48 hours and that red blood cells were lysed, we excluded myeloid lineages (mainly composed of neutrophils), erythrocytes, and platelets from the analysis and focused on the T cell subsets, including naïve, proliferating, CM, and EM T CD4^+^ or CD8^+^ cells. After identifying these clusters, we performed a differential expression analysis on the normalized data (patient versus control) using an analysis of variance (ANOVA) model for each CD4^+^ and CD8^+^ T cell with Partek Flow. Up- and down-regulated genes were selected on the basis of the false discovery rate (FDR)–adjusted *P* value (≤0.05) and the log_2_ fold change (<−1 or >1). Gene-specific pathway analysis was performed using Metascape with a *q* value of <0.05 in the Gene Ontology (GO) biological processes database (*70*). Raw data (FASTQ files) are available at the National Center for Biotechnology Information’s Sequence Read Archive under the ArrayExpress accession E-MTAB-13124.

### Western blotting

Western blot analysis was performed on 10^6^ HDFs or Jurkat cells. Frozen cell pellets were thawed on ice before the addition of ice-cold M-PER lysis buffer (Thermo Fisher Scientific, M-PER Lysis Buffer, #78503, and Halt Protease and Phosphatase Inhibitor Cocktail, #78876). Cells were lysed on ice for 15 min, shaking and occasional vortexing, and then centrifuged for 15 min at 16,000*g*, when the supernatant was recovered. Thirty micrograms of each sample was mixed with loading buffer and heated for 30 min at 60°C before loading (20 to 30 μl) on a 7.5% Mini-PROTEAN TGX Precast Protein Gels (Bio-Rad Laboratories, Hercules, CA). Electrophoresis was conducted at 100 V for 1 hour and 30 min, before transfer to a polyvinylidene fluoride membrane using a Trans-Blot Turbo Transfer system (Bio-Rad Laboratories, Hercules, CA) according to the manufacturer’s instructions. Membranes were blocked in tris-buffered saline, Tween 20 (0.05%), and nonfat milk (5%) for 1 hour before incubation with an anti-IP3R3 monoclonal antibody (mouse IgG; #610312, BD Transduction Laboratories, Franklin Lakes, NJ) used at a 1:1000 dilution and anti-IP3R2 monoclonal antibody (mouse IgG; #sc-398434, Santa Cruz Biotechnology, Dallas, TX) used at a 1:100 dilution, overnight at 4°C. Secondary antibodies [goat anti-mouse IgG [H^+^L]–horseradish peroxidase (HRP) conjugates; #1706516, Bio-Rad Laboratories, Hercules, CA] were used for 1 hour at room temperature at 1:5000 dilution. Signals were revealed using the SuperSignal West Femto Maximum Sensitivity Substrate (Thermo Fisher Scientific, Waltham, MA, USA), and detection was performed using the ChemiDoc XRS^+^ system (Bio-Rad Laboratories, Hercules, CA). Anti-calnexin (rabbit IgG; ab22595, Abcam, Cambridge, UK), used at a 1:1000 dilution, was applied for 1 hour at room temperature. Secondary antibodies (goat anti-rabbit IgG [H^+^L]–HRP conjugates; #1721019, Bio-Rad Laboratories, Hercules, CA) were used for 1 hour at room temperature at 1:5000 dilution. Signals were revealed using the Clarity Western ECL (Bio-Rad Laboratories, Hercules, CA), and detection was performed using the ChemiDoc XRS^+^ system (Bio-Rad Laboratories, Hercules, CA).

### Proteomic analysis

Total proteins were extracted from PBMC pellets and HDFs in, respectively, 3 or 5% SDS and 10 mM tris (pH 6.8). Protein concentrations were determined using the DC protein assay kit (Bio-Rad Laboratories, Hercules, CA) according to the manufacturer’s instructions. The single-pot solid-phase enhanced sample preparation (SP3) protocol was carried out manually as previously described (*71*). Briefly, 50 μg of each protein extract was reduced with 12 mM DTT and alkylated using 40 mM iodoacetamic acid. A mixture of hydrophilic and hydrophobic magnetic beads was used to clean up the proteins at a ratio of 20:1 beads:proteins (Sera-Mag Speed beads, Thermo Fisher Scientific, Waltham, MA). After addition of acetonitrile (ACN) to a final concentration of 50%, the beads were allowed to bind to the proteins for 18 min. Proteins-beads mixtures were washed twice with 80% ethanol and once with 100% ACN. The proteins-beads complexes were digested with a mixture of trypsin:LysC (Promega, Madison, WI) at a 1:20 ratio overnight at 37°C. Extracted peptides were cleaned-up using automated C18 solid phase extraction on the Bravo AssayMAP platform (Agilent Technologies, Santa Clara, CA). Nano–liquid chromatography–tandem mass spectrometry (NanoLC-MS/MS) analyses were performed on a nanoAcquity Ultra Performance LC device (Waters Corporation, Milford, MA) coupled to a quadrupole Orbitrap mass spectrometer (Q-Exactive HF-X, Thermo Fisher Scientific, Waltham, MA). Chromatographic separation was conducted with the following gradient of solvent B: from 1 to 8% over 2 min, from 8 to 35% over 77 min, and from 35 to 90% over 1 min. A top 20 method was used with automatic switching between MS and MS/MS modes to acquire high-resolution MS/MS spectra. Samples were injected using a randomized injection sequence, and two solvent blank injections were performed after each sample to minimize carryover. NanoLC-MS/MS data were interpreted to do label-free extracted ion chromatogram–based differential analysis using MaxQuant (version 1.6.14.0) (*72*). Peaks were assigned with the Andromeda search engine against a database extracted from UniProtKB-SwissProt (14-09-2020; 20,388 sequences, taxonomy ID 9606) with trypsin/P specificity. The minimal peptide length required was seven amino acids, and a maximum of one missed cleavage was allowed. Methionine oxidation and acetylation of the protein’s N termini were set as variable modifications and cysteine carbamidomethylation as a fixed modification. No “match between runs” was performed between the samples. The maximum FDR was 1% at peptide and protein levels with the use of a decoy strategy. Label-free quantification intensity values were exported and used for further statistical analysis. The complete proteomic dataset has been deposited into the ProteomeXchange Consortium via the PRIDE partner repository with the dataset identifier PXD038284 (*73*).

### Differential RNA and protein expression

Volcano plots were generated with R using the ggplot2 library (*74*). Pathway analyses of differentially expressed proteins were performed using Metascape (*70*) based on differentially expressed gene lists selected with an adjusted *P* value < 0.05 and a minimal fold change of 2 and 1.5 for HDFs and PBMCs, respectively (*70*). GSEA was performed with the GSEA software from the Broad Institute (http://software.broadinstitute.org/gsea/index.jsp) (*75*, *76*). The gene sets from the human GO database were used to compute the enrichment of differentially expressed proteins. The normalized enrichment score, nominal *P* value, and FDR *q* value were assessed using the gene_set mode with 1000 permutations.

### Confocal microscopy

HDFs were seeded on glass coverslips (VWR, Radnor, PA), precoated with fibronectin bovine plasma (Merck, Burlington, MA). In some conditions, cells were labeled with 200 nM MitoTracker Deep Red (Invitrogen, Carlsbad, CA) in DMEM for 30 min at 37°C and then washed with PBS-Ca^2+^-Mg^2+^. All subsequent incubations were performed at room temperature in the dark, and washes were performed in PBS without Ca^2+^ and Mg^2+^. Fixation was performed with 4% paraformaldehyde (PFA) solution (Electron Microscopy Science, Hatfield, PA) for 20 min, followed by quenching with NH_4_Cl 50 mM for 1 min. Cells were washed and permeabilized with 0.5% NP-40 solution for 5 min and then washed three times. Blocking was performed with PBS-Ca^2+^-Mg^2+^ and 2% BSA (Merck, Burlington, MA). Cells were incubated for 1 hour and 30 min with either anti-IP3R3 antibody (mouse IgG; 610312, BD Biosciences; used at 1:500 dilution, Franklin Lakes, NJ) or anti-IP3R2 monoclonal antibody (mouse IgG; SC398434, Santa Cruz Biotechnology, Dallas, TX; used at 1:250 dilution) in PBS-Ca-Mg, 2% BSA, and, in some conditions, with anti-calnexin polyclonal antibody (rabbit IgG; PA5-34754, Thermo Fisher Scientific, Waltham, MA; used at 1:200 dilution) in PBS-Ca^2+^-Mg^2+^ and 2% BSA. Cells were then washed three times. Goat anti-mouse IgG Alexa Fluor 488 and goat anti-rabbit IgG Alexa Fluor 555 (A11029 and A21429, respectively; Thermo Fisher Scientific, Waltham, MA; used at 1:500 dilution) secondary antibodies were incubated for 45 min in PBS-Ca^2+^-Mg^2+^ and 2% BSA. Cells were washed three times and mounted on glass slides with Fluoromount-G with 4′,6-diamidino-2-phenylindole (Invitrogen, Carlsbad, CA). Image acquisition was performed on a Zeiss LSM 800 confocal microscope (Zeiss, Oberkochen, Germany) with suitable lasers, optics, and GaAsP PMT detectors for the required fluorescence. Images were acquired at 70.5 nm/pixel. Image analysis and processing were performed with FIJI and CellProfiler (www.cellprofiler.org) softwares. Oversaturation of each fluorescence channel was used to define regions of interest to isolate single cells in FIJI. For each of these cells, CellProfiler (www.cellprofiler.org) pipeline was used to normalize signal, remove background, identifying primary objects using Otsu thresholding, and merge objects to analyze overlaps, for each fluorescence channel. Data of area, intensities, and quantity of overlaps were used for the analysis.

### Electron microscopy

HDFs were seeded on 35-mm glass-bottom dishes. Samples were chemically fixed right after photonic acquisition with 2.5% glutaraldehyde and 2.0% PFA in 0.1 M NaCac buffer (pH 7.4), kept for 48 hours at 4°C, and then washed three times with 0.1 M NaCac buffer (pH 7.4), followed by a second fixation with 0.05% malachite green and 2.5% glutaraldehyde in 0.1 M NaCac buffer (pH 7.4) for 30 min in an ice bath. Subsequently, samples were postfixed in 1% OsO_4_ to 0.8% K_3_Fe (CN)6 in 0.1 M NaCac buffer (pH 7.4) in an ice bath for 50 min and then washed two times with 0.1 M NaCac (the buffer was also kept in an ice bath to avoid thermal changes). The samples were then incubated in 1% aqueous tannic acid solution for 25 min in an ice bath and lastly washed five times with distilled water. Samples were then kept in 1% uranyl acetate overnight at 4°C and sheltered from light. Samples were serially dehydrated with ethanol solutions (25, 50, 70, 95, and 100%). Subsequently, samples were incubated in a serial resin-ethanol 100% solution (1:3; 1:1; and 3:1), ending with an incubation in 100% Epon resin three times 1 hour at room temperature. Samples were then allowed to polymerize in an oven at 60°C for 48 hours. The resin block was trimmed by ultramicrotomy, i.e., 90-nm-thick sections were collected and placed in 200 mesh cupper grids. The transmission electron microscopy (TEM) dataset was acquired with a Hitachi 7500 TEM, with 80-kV beam voltage, and the 8-bit images were obtained with a Hamamatsu camera C4742-51-12NR (Hamamatsu, Bridgewater, NJ). Image analysis and processing were performed with FIJI [Fiji: an open-source platform for biological image analysis, PubMed (nih.gov)] and CellProfiler. For contact site areas at 10 nm, mitochondria masks were expanded for a fixed number of pixels representing 10 nm. When an ER object was recognized, the area of the overlapping region between ER and expanded mitochondria was measured.

### Flow cytometry

In addition to routine clinical flow cytometry performed at each hospital diagnostic laboratory, more in-depth flow cytometry was performed on the whole blood of patients 2 and 4, both in diagnostic (*77*–*79*) and research settings. Briefly, diagnostic flow cytometry was performed as follows. Whole blood was used, and staining for cell surface markers was performed after red cell lysis by the TQ-prep and ImmunoPrep Reagent System (Beckman Coulter Life Sciences, Indianapolis, IN). To define total major lymphocyte subsets, the Beckman Coulter panels Tetra-1 (CD45-FITC/CD3-PC5/CD4-RD1/CD8-ECD) and Tetra-2 (CD45-FITC/CD3PC5/CD56/16-RD1/CD19-ECD) (Beckman Coulter Life Sciences, Indianapolis, IN) were used. Absolute lymphocyte values were calculated using a single-platform method based on precalibrated Flow-Count fluorescent microspheres (Beckman Coulter Life Sciences, Indianapolis, IN). The following monoclonal antibodies were used to define T cell subsets in patient 2: CCR7-FITC (#150503, BD Biosciences), CD3-PB (#UCHT1, Beckman Coulter Life Sciences, Indianapolis, IN), CD4-PC7 (#SFCI12T4D11, Beckman Coulter Life Sciences, Indianapolis, IN), CD8-APC (#SFCI12T4D11, Beckman Coulter Life Sciences, Indianapolis, IN), CD45RA-ECD (#2H4, Beckman Coulter, Beckman Coulter Life Sciences, Indianapolis, IN), and CD45RO–phycoerythrin (PE) (#UCHL1, Beckman Coulter, Beckman Coulter Life Sciences, Indianapolis, IN). The following monoclonal antibodies were used to define B cell subsets: CD19-APCR700 (#564977, BD), CD27-PE (#MT271, BD), CD21-FITC (#BL13, BD), CD24-BV421 (#ML5, BD), CD38-PC7 (#HIT2, BD), IgD-PECF594 (#IA62, BD), and IgM-APC (#G20-127, BD). Samples were analyzed using a Navios 10 colors/3 lasers flow cytometer (Beckman Coulter Life Sciences, Indianapolis, IN), and data analysis was performed with Kaluza software (Beckman Coulter Life Sciences, Indianapolis, IN). T cell subsets were defined as naïve (CD45RA^+^CCR7^+^), CM (CD45RA^−^CCR7^+^), EM (CD45RA^−^CCR7^−^), and TEMRA (CD45RA^+^CCR7^−^) cells among the CD4^+^ and the CD8^+^ T cells. B cell subsets were defined among CD19^+^ B cells as naïve (CD27^−^IgD^+^), switched memory (CD27^+^IgD^−^, IgM^−^), unswitched memory (CD27^+^IgD^+^, IgM^+^), transitional (CD24^++^CD38^++^), plasmablasts (CD27^+^CD38^+++^), and CD21/CD38low (CD21low, CD38low).

The 40 color flow cytometry of patient 2 samples was performed using a previously established protocol (table S2) (*80*). Briefly, 250 μl of whole blood was washed twice with 2 ml of 1× PBS, spun at 350*g* for 5 min at room temperature. The samples were then stained with 1 ml of 1:1000 diluted Live/Dead Blue for 20 min at room temperature. Samples were then washed with 2 ml of FACS buffer (PBS 1×, FBS 0.5%, EDTA, 2 mM, and Hepes 10 mM) and then collected by centrifugation at 300*g* for 5 min at room temperature. Five microliters of Human TruStain FcX and 5 μl of True-Stain Monocyte Blocker were added to each in the control and panel, vortexed, and incubated for 5 min. The mix of 40 antibodies was added in three batches (see table S2 for details). After staining for 30 min on ice, 3 ml of red blood cell lysis buffer (Versalyse, Beckman Coulter Life Sciences, Indianapolis, IN) was added to each sample. After 10 min of incubation at room temperature, the cells were immediately centrifuged at 300*g* for 5 min at 4°C. Samples were washed twice with 2 ml of FACS buffer, centrifuged, and resuspended in 300 μl of FACS buffer. The resuspended material was then analyzed using a Cytek Aurora 5L spectral flow cytometer (Cytek Biosciences, Fremont, CA). A total of 300,000 CD45-positive live cells were acquired. Whole blood samples were analyzed by using the OMIQ software from Dotmatics (www.omiq.ai, www.dotmatics.com). Downsampling was performed on 300,000 CD45^+^ cells. In this composite file, cells were pregated as follows: singlets, live, time, and CD45^+^.

### T cell proliferation

The proliferation of T cells upon stimulation with anti-CD3 was measured by the Click-iT EdU assay. PBMCs of patient 4 or healthy controls were first seeded at 1 × 10^6^ cells/ml in RPMI 1640 medium supplemented with l-glutamine and 5% human AB serum in sterile, flat-bottom, 48-well culture plates that contained either medium alone or with the addition of anti-CD3 (12 μg/ml; HIT3a, BD Biosciences, Franklin Lakes, NJ) antibody or anti-CD3 (12 μg/ml) plus anti-CD28 (0.1 mg/ml; CD28.2, BD Biosciences, Franklin Lakes, NJ) antibodies. After 72 hours of incubation, alkyne-modified EdU (Invitrogen, Carlsbad, CA) was then added to each well, where it incorporated into the synthesizing DNA during a second incubation of 18 to 24 hours. After the second incubation, the cells were stained with anti-CD45 APC-H7 (2D1, BD Biosciences, Franklin Lakes, NJ) and anti-CD3 PerCP-Cy5.5 (SK7, BD Biosciences, Franklin Lakes, NJ) to label the T cells. After fixation and permeabilization, via a copper-catalyzed click chemistry reaction, the incorporated EdU was then covalently labeled by incubating the cells with azide-modified Alexa Fluor 488 (Invitrogen, Carlsbad, CA) for 30 min. Cells incubated in medium only but without the second incubation of EdU were treated with azide-modified Alexa Fluor 488 serve as a control for the Click-iT reaction. After washing, the cells were then analyzed. Data from a minimum of two independent experiments were analyzed using FlowJo (BD Biosciences, Franklin Lakes, NJ).

### Statistics

Statistical analyses were performed using GraphPad Prism 9.5.0 (GraphPad Software, San Diego, CA). The applied tests and associated *P* values are provided for each figure in its legend.
